# Identifying High Ionic Conductivity Compositions of Ionic Liquid Electrolytes Using Features of the Solvation Environment

**DOI:** 10.1021/acs.jctc.4c01441

**Published:** 2025-02-11

**Authors:** Amey Thorat, Ashutosh Kumar Verma, Rohit Chauhan, Rohan Sartape, Meenesh R. Singh, Jindal K. Shah

**Affiliations:** † School of Chemical Engineering, Oklahoma State University, Stillwater, Oklahoma 74078, United States; ‡ Department of Chemical Engineering, University of Illinois at Chicago, Chicago, Illinois 60608, United States

## Abstract

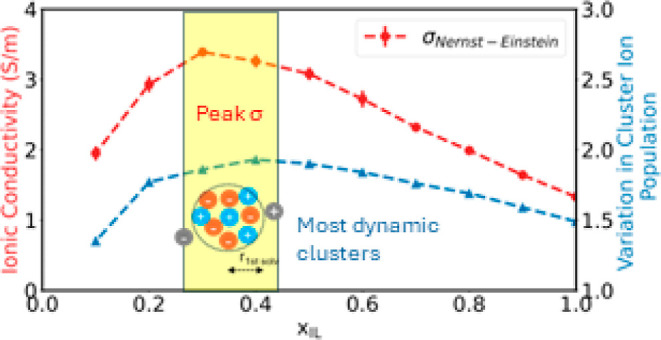

Binary mixtures of ionic liquids with molecular solvents are gaining interest in electrochemical applications due to the improvement in their performance over neat ionic liquids. Dilution with suitable molecular solvents can reduce the viscosity and facilitate faster diffusion of ions, thereby yielding substantially higher ionic conductivity than that for a pure ionic liquid. Although viscosity and diffusion coefficients typically behave as monotonic functions of concentration, ionic conductivity often passes through a peak value at an optimum molar ratio of the molecular solvent to the ionic liquid. The ionic conductivity maximum is generally explained in terms of a balance between the ease of charge transport and the concentration of the charge carriers. In this work, fluctuation in the local environment surrounding an ion is invoked as a plausible explanation for the ionic conductivity mechanism with a binary mixture of 1-ethyl-3-methylimidazolium tetrafluoroborate and ethylene glycol as an example. The magnitude of the dynamism in the local environment is captured by measuring the spatial and temporal features of the solvation environment. Standard deviation in the number of ions in the solvation environment serves as a spatial feature, while the cage correlation lifetimes for oppositely charged ions within the first solvation shell serve as a temporal feature. Large standard deviations in the cluster ion population and short cage correlation lifetimes are indicators of highly dynamic ionic environment at the molecular level and consequently yield high ionic conductivity. Such compositions were found to be in good agreement with the optimum ionic liquid mole fractions obtained through experimental measurement. Short cage correlation lifetimes enable the identification of optimum mixture compositions using simulation trajectories significantly shorter than those required to implement the Nernst–Einstein or Einstein formalisms for calculating ionic conductivity. We validated the applicability of this approach across force fields and in six ionic liquid-molecular solvent electrolytes formed with combination of cations, anions, and solvents. We offer a computationally efficient approach of screening ionic liquid-molecular solvent binary mixture electrolytes to identify molar ratios that yield high ionic conductivity.

## Introduction

Low volatility, high thermal and electrochemical stability, and high ionic conductivity make ionic liquids attractive candidates for use as electrolytes in energy storage^1−3^ and other electrochemical applications.^4−6^ The presence of polar and nonpolar moieties on the constituent ions imparts compatibility with a large number of ionic^7^ and molecular solvents.^8^ This provides flexibility to tailor the physicochemical properties of the binary mixtures of molecular solvents and ionic liquids toward specific applications. Dilution with suitable molecular solvents can reduce viscosity^9,10^ and improve processability compared to neat ionic liquids. Many ionic liquids are also known to be toxic;^11−13^ hence, mixing them with more benign molecular solvents can help mitigate the overall environmental impact. Some ionic liquids can be expensive; hence, dilution also offers significant cost benefits. However, the most significant advantage of mixing ionic liquids and molecular solvents pertinent to electrochemical processes is the boost in ionic conductivity.^14,15^ The magnitude of improvement in conductivity achieved by mixing molecular solvents is determined by the favorable or unfavorable interactions between the ions and the solvent molecules. This necessitates systematic inquiry into the relation among the molecular structure, ion-organization, ion–ion interactions, and trends in the ionic conductivity of binary mixtures of ionic liquids and molecular solvents.

The increase in the ionic conductivity of binary mixture electrolytes is broadly explained based on the reduction in viscosity, faster ion diffusion, and optimum number of charge carriers. However, ionic conductivity in binary mixtures is a complex phenomenon and is influenced by multiple factors such as size and charge on the ions, polarity and dielectric strength of the solvent, viscosity, hydrogen bonding strength, ion association, etc. Theoretical frameworks dedicated to capturing the relationship between ionic conductivity and some of these factors have been previously attempted.^16−19^ Several other works have investigated the impact of these factors through experiment^14,20−23^ and computation.^24−27^ For example, Matsumoto and co-workers^24^ implemented molecular dynamics (MD) simulations to study the effect of ion association in mixtures of 1-butyl-3-methylimidazolium bis(trifluoromethylsulfonyl)imide in 22 unique solvents. They compared the ion shielding effect due to various solvents based on the dielectric constants over a range of ionic liquid concentrations. McDaniel and Son^26^ resolved the net ionic conductivity into the contributions due to the self-and cross correlations between ions in binary mixtures of 1-butyl-3-methylimidazolium tetrafluoroborate ([BMIM][BF_4_]) in dichloroethane, acetone, acetonitrile, and water and discussed in detail the effects of solvent polarity on ion-correlation and contribution to conductivity. Interactions among polar and nonpolar domains of the ionic liquid and molecular solvent also play a significant role in determining the organization of ions. These can induce a certain degree of mesoscale organization in the ionic liquid structure that significantly impacts the bulk properties.^28−31^ However, most works limit the discussion concerning the changes in the solvation environment or ion organization to radial or spatial distribution functions. Radial distribution functions and coordination numbers provide an overall picture of the system averaged over time, space, and the number of charge carriers. On the other hand, techniques that focus on instantaneous and local environment such as cluster analysis^32−34^ and cage correlations^35−38^ can be more useful to probe into the nonhomogeneity within the system or study transport properties such as ionic conductivity. Regardless, works dedicated to the study of the evolution of the solvation environment in binary ionic mixture electrolytes and the influence on the ionic conductivity are limited.

Most computational studies implement a diffusion-based Nernst–Einstein approach for the estimation of ionic conductivity. This approach uses self-diffusion coefficients of the ions and is more suitable for dilute systems with negligible ion–ion interactions. Pure ionic liquids composed entirely of ions are far from dilute, and charges on the ions induce strong electrostatic interactions among the ions. As a result, deviation between the experimentally measured and predicted Nernst–Einstein conductivity values are common. On the other hand, the rigorous Einstein formalism offers much better quantitative predictions by considering all ion–ion interactions. However, it is computationally expensive to employ the Einstein approach for high-throughput screening to identify compositions of ionic liquid–molecular solvent mixtures, yielding high ionic conductivity. This is primarily due to the need for either multiple^39^ or longer^40^ trajectories to allow for sufficient sampling and is consequently a less popular choice. Avula et al.^41^ have described an elaborate force field optimization strategy for the prediction of ionic liquid properties including ionic conductivity. A combination of an optimized force field and Einstein formalism can yield the most accurate values, but such rigorous methods are prohibitively expensive in screening applications where the main interest is to identify the mixture compositions with high conductivity as opposed to the exact values. Some other approaches to estimate ionic conductivity include the modified Green–Kubo method^42^ or an approximation proposed by France-Lanord and Grossman.^43^ All approaches are sensitive to force fields, and small changes in the values of bonded or nonbonded parameters such as Lennard-Jones (LJ) parameters or atomic charges can lead to substantial deviation between the predicted and experimental values or the overall trend in ionic conductivity.^44^ Differences between predicted and experimental values tend to be large, especially with generic, nonoptimized force fields. Regardless, these approaches can provide helpful qualitative estimates on ionic conductivity and the overall trend and thereby guide the design of efficient experiments.

Commonly investigated nonaqueous molecular solvents, for applications of ionic liquids in electrochemical processes, include alcohols,^27,45,46^ acetonitrile,^47^ ethylene and propylene carbonates, etc.^48,49^ Systems containing Li^+^ and Na^+^ compounds have been extensively studied due to their application in batteries but are beyond the scope of the current work. The molecular solvent of interest in this work is ethylene glycol (EG). A solution of potassium hydroxide (KOH) in EG is deployed as a nonaqueous, hydrogen bonding capable, reactive solvent for capturing CO_2_ in the migration-assisted moisture-gradient (MAMG) electrochemical process.^50^ It was observed that the overall energy efficiency of the process could be improved by about 50% by increasing the ionic conductivity alone.^51^ Further improvement in the process efficiency is being sought through the addition of ionic liquids such as those based on imidazolium cations into the mixture since they offer both high ionic conductivity and high molar CO_2_ solubility.^7,52,53^ Hence, a mixture of 1-ethyl-3-methylimidazolium tetrafluoroborate [EMIM][BF_4_] in EG was selected as a prototypical system for investigation in this work.

The first part of this work uses MD simulations to estimate the ionic conductivity of binary mixtures of [EMIM][BF_4_] in EG at mole fractions between 0.1 and 1.0 at 298 K and 1.0 bar using the popular Nernst–Einstein and Einstein approaches and compares the predictions with the experimental measurements. Next, ion–ion correlations are utilized to resolve the net ionic conductivity into contributions due to self-correlations and cross correlations between cation–cation, cation–anion, and anion–anion. Radial distribution functions are then used to examine any differences in the average distribution of ions around cation, anion, and solvent species as a function of concentration. Trends in cluster analysis and cage correlation lifetimes are obtained to provide qualitative and quantitative insight into the evolving ion-demographics and ion-dynamics within the first solvation shell as a function of mixture composition. [EMIM][BF_4_] in EG is also simulated using two other force fields to test the effect of the force field on the solvation shell dynamics. The results obtained using the longer trajectories using the *NVT* ensemble are compared with those obtained by using shorter trajectories from the *NPT* ensemble. With sufficient agreement between the results obtained using the *NPT* ensemble, six other systems containing binary mixtures of ionic liquid and molecular solvents are analyzed, and trends in solvation shell dynamics are compared against Nernst–Einstein conductivity and reported experimental values. The composition with the most dynamic solvation shell was observed to be in close agreement with the ionic liquid mole fractions that exhibit the peak ionic conductivity.

## Methods

### Experiment

#### Materials and Sample Preparation

EG (99% purity) was obtained from Sigma-Aldrich, while [EMIM][BF_4_] (99.99% purity) was purchased from Iolitec GmbH, Germany. EG was dried in a vacuum oven at 60 °C for 12 h prior to the use in an experiment. Samples of ionic liquid mole fractions (*x*_IL_) ranging from *x*_IL_ = 0.1 to *x*_IL_ = 1.0, spaced at 0.1 each, were prepared by dissolving appropriate amounts of [EMIM][BF_4_] in EG.

#### Conductivity Measurements

The ionic conductivity of the samples was measured using a conductivity meter (Orion Star A212 Conductivity Benchtop Meter, Thermo Scientific, USA) and a conductivity probe (Orion 013005MD, Thermo Scientific, USA) at ambient pressure. All of the measurements were conducted at 298 K controlled by a thermostat (PT100 Probe, Chemglass Life Sciences, USA).

### Simulation

#### Force Fields

The virtual site force field for ionic liquids (VSIL)^54^ was used to represent [EMIM][BF_4_]. EG was modeled using the OPLS-DES force field.^55^ Both force fields are based on the nonpolarizable OPLS all-atom force field^56^ and have been shown to reproduce thermophysical properties of a large number of imidazolium-based ionic liquids.^54^ These force fields estimate the total energy based on the bonded and nonbonded interactions among the atoms. The intramolecular bonded interactions are further resolved into harmonic stretching, harmonic angle bending, and dihedrals, while Coulombic and LJ terms contribute toward the nonbonded interactions. The total energy is estimated using the following equations
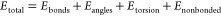
1
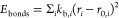
2
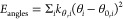
3
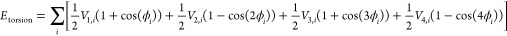
4
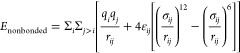
5where *k*_θ_ and *k*_b_ represent the force constants for angle bending and bond stretching,respectively, while the Fourier coefficients for torsion in dihedral interactions are denoted by *V*_*i*_. *r*_0,*i*_ and θ_0,*i*_ signify the nominal values for the bond length and angle, respectively. σ and ε refer to the size and energy parameters for the 12–6 LJ interactions, respectively. Electrostatic charges are denoted by *q*. Geometric combination rules were used for the estimation of LJ parameters for unlike interactions where  and . All 1–4 nonbonded interactions were scaled using a factor of 0.5. A cutoff distance of 16 Å was used to apply the tail corrections for electrostatic and LJ interactions. The PME method was used to compute the long-range electrostatic energy.

#### *NVT* MD Simulations

MD simulations were carried out using GROMACS 2018^60−67^ in two stages. The first stage determined the system volume for different ionic liquid concentrations using the isothermal–isobaric (*NPT*) ensemble, while the second stage focused on the estimation of ionic conductivity using the canonical (*NVT*) ensemble with volume determined from the density obtained from *NPT* simulations. Ten systems, each containing a total of 500 molecules with a relevant number of [EMIM][BF_4_] ion pairs and EG molecules, were prepared to represent ionic liquid concentrations with mole fractions from 0.1 to 1.0, spaced at a mole fraction of 0.1. For each system, an initial configuration was generated using PACKMOL^68^ by packing the components in a cubic box, the volume for which was estimated using ideal mixing behavior. Periodic boundary conditions were enforced along the *x*-, *y*-, and *z* axes. A steepest descent energy minimization was carried out to remove any high energy configurations or particle overlaps. The system was then subjected to a high-temperature annealing protocol for 2 ns, in which the system temperature was raised to 498 K for about 300 ps, after which the system temperature was lowered gradually to 298 K and maintained at this temperature. An *NVT* and *NPT* equilibration run ensued, each for 10 ns in which the temperature was controlled with velocity-rescale temperature coupling^69^ (τ_*T*_ = 1.0 ps), while Berendsen pressure coupling^70^ (τ_*p*_ = 1.0 ps) was implemented to maintain pressure at 1.0 bar. The final production run of 20 ns was conducted in the *NPT* ensemble, from which the box volume was extracted for use in the second stage.

In the second stage, an initial configuration for each of the systems was generated by packing a total of 500 molecules containing a relevant number of EG and [EMIM][BF_4_] molecules in a cubic box for a given mole fraction of the ionic liquid, with volume obtained from the first stage. Energy minimization, high-temperature annealing, and *NVT* equilibration were carried out as outlined for the first stage. The final production run was performed in the *NVT* ensemble for 100 ns to allow sufficient sampling of the trajectory for estimation of ionic conductivity using the Einstein formalism as documented in our previous work.^40^ Temperature was maintained using the Nosé–Hoover thermostat^71^ (τ_*p*_ = 1.0 ps). Three sets of data were collected using distinct initial configurations for every ionic liquid mole fraction to obtain statistical consistency.

### Ionic Conductivity Estimation

#### Nernst–Einstein Formalism

The self-diffusion coefficients for the cation (*D*_+_) and the anion (*D*_–_) were calculated from the production run trajectory, where the mean-square displacement (MSD) evolved as a linear function of time. An ensemble average was calculated by averaging over different time origins to efficiently capture the ion displacements. Self-diffusion coefficients were calculated by using eq 6
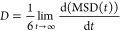
6The MSD, for a given time *t*, can be calculated by tracking the positions of individual particles
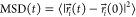
7where the position of the *i*^th^ particle at time *t* is given by , while  indicates the position of the particle at the time origin. The diffusion coefficients of the cation and anion were then used to estimate the Nernst–Einstein (σ_NE_) conductivity using eq 8
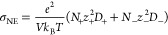
8where *e* is charge on an electron, *k*_B_ is the Boltzmann constant, *V* is the system volume, *T* is the temperature, and *z*, *N*, and *D* are charge, number, and diffusion coefficients of cations (*D*_+_) and anions (*D*_–_) respectively.

#### Einstein Formalism

In the Einstein formalism, all ion–ion interactions are taken into account. Equation 9 was used to estimate the overall ionic conductivity
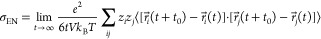
9Statistical uncertainty of the Einstein conductivity estimates was improved by employing time-origin shifting. The product term was plotted as a function of time from which the slope obtained from the first 2 ns was used to estimate the Einstein conductivity.^40^

### Ion–Ion Correlations

Ion–ion correlations were evaluated to estimate the contributions due to cation–cation, anion–anion, and cation–anion interactions. These correlations were calculated using the Einstein equation (eq 9) by manipulating *i* and *j* values (*i* ≠ *j)* to include only cations, only anions, and both cations and anions, respectively. Cation–cation and anion–anion self-correlations were estimated by using *i* = *j*.

### *NPT* Screening

Estimates of transport properties using the *NPT* ensemble can be impacted due to the effect of wrapping algorithms;^72^ however, such effects are likely to be insignificant in sufficiently large systems as those considered in this work. We tested if optimum mixture compositions could be predicted by carrying out the cluster analysis and ionic conductivity estimation using production run trajectories generated during the volume estimation stage of the workflow described earlier using the *NPT* ensemble. By bypassing the need to calculate long 100 ns trajectories in the *NVT* ensemble, this method offers qualitative screening of a wider range of mixtures in a faster and computationally efficient manner compared to the two-stage workflow described earlier. The main focus of this work is to identify highly conductive mixture compositions rather than the actual values for conductivity. Since both the Nernst–Einstein and Einstein approaches yield peak conductivity at similar mole fractions (see below), we resort to the computationally efficient Nernst–Einstein approach for estimation of the conductivity trend.

### Other Ionic Liquid—Molecular Solvent Binary Mixture Electrolytes

To validate whether the fluctuations in the local environment reveal trends in ionic conductivity in other ionic liquid and molecular solvent combinations, we tested mixtures of [EMIM][BF_4_] in two additional molecular solvents: acetonitrile and ethanol. To test across ionic liquids containing different cations, we analyzed binary mixtures of [BMIM][BF_4_] in EG. Systems containing different anions were analyzed using binary mixtures of ionic liquids based on 1-ethyl-3-methylimidazolium thiocyanate [EMIM][SCN] in EG, 1-ethyl-3-methylimidazolium triflate [EMIM][TfO] in EG, and 1-ethyl-3-methylimidazolium dicyanamide [EMIM][DCA] in EG. The OPLS force fields for the solvents were obtained from LigParGen,^73−75^ while those for the ionic liquids were used from the OPLS-based force fields developed by Doherty et al.^54,55^ A list of force fields used is available in Table S2 in the Supporting Information.

## Results and Discussion

### Ionic Conductivity

**Figure 1:**
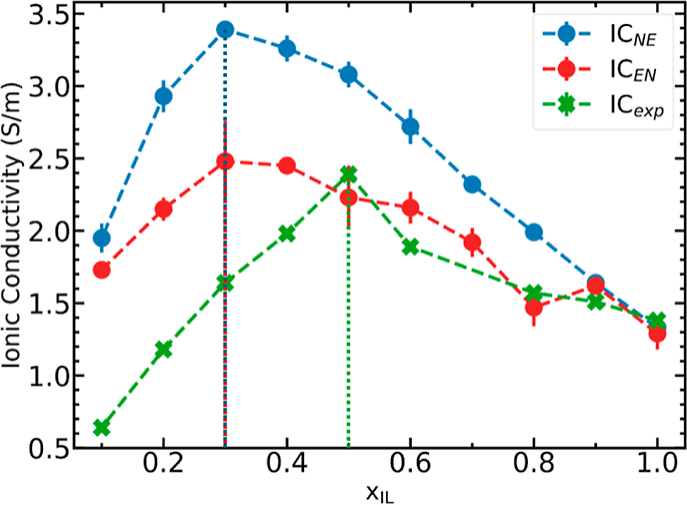
Trends in ionic conductivity as a function of the [EMIM][BF_4_] concentration at 298 K. Nernst–Einstein conductivity (IC_NE_) and Einstein conductivity (IC_EN_) compared with experimental values (IC_exp_). Vertical lines indicate mole fractions with peak conductivity values.

Figure 1 represents a comparison between the predicted ionic conductivity using Nernst–Einstein and Einstein formalisms and the experimentally measured values. Both approaches predict a nonmonotonic trend in ionic conductivity as a function of the ionic liquid concentration. Although the actual predicted values are different, both approaches indicate peak conductivity at *x*_IL_ = 0.3 as against *x*_IL_ = 0.5 measured from experiment. Predicted ionic conductivity values are higher than the measured values at all compositions, except for pure [EMIM][BF_4_], with larger deviations in solvent-rich systems. The Nernst–Einstein conductivity estimates are overpredicted, higher than both experimental and Einstein conductivity values, an observation consistent with theory, given that the diffusion-based Nernst–Einstein approach assumes noninteracting ions. The Einstein approach offers better accuracy on the actual values of ionic conductivity, albeit at the higher computational cost Both Nernst–Einstein and Einstein formalisms peak at *x*_IL_ = 0.3, suggesting that both approaches are of comparable accuracy in identifying the optimum mixture compositions. This supports the use of the Nernst–Einstein formalism for qualitative screening applications.

Lower ionic conductivity in the dilute regions is expected due to fewer ions available for charge transport as compared to the ionic liquid-rich systems. Formation of associated ion-pairs can result in a further decrease in the number of net charge carriers. These effects yield lower ionic conductivity even though the self-diffusion coefficients for the ions are substantially higher than those in more concentrated systems. On the other hand, in mixtures with higher mole fractions of ionic liquid, the conductivity tends to decrease due to strong ion–ion interactions, increased viscosity, and hindered ion diffusion. Thus, the ionic conductivity tends to decrease in mixtures at either end of the ionic liquid concentration spectrum, with values peaking at mixing ratios that provide a large number of charge carriers and sufficiently fast ion diffusion that facilitates rapid charge transport.

### Ion–Ion Correlations

**Figure 2:**
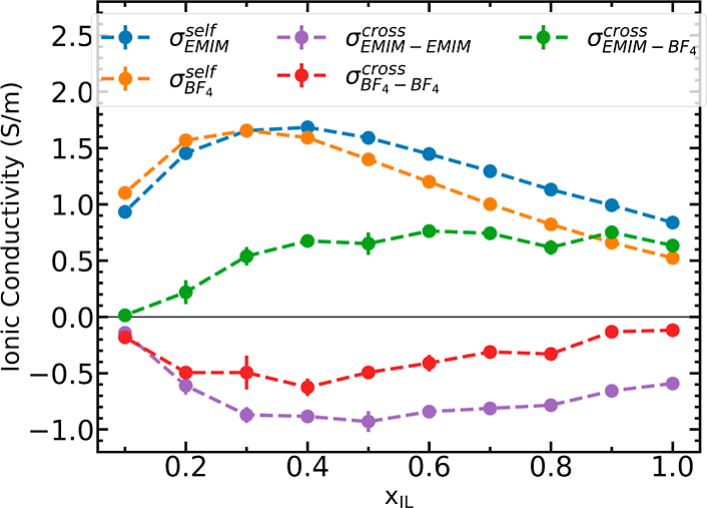
Contribution to ionic conductivity from self-correlation of cations (blue) and anions (orange); and cross correlation between cation–cation (purple), anion–anion (red), and cation–anion (green).

Understanding the correlation between cations and anions is important to resolve their contribution toward ionic conductivity. Correlated movement of like charged ions boosts ionic conductivity because it enhances net charge transport. On the other hand, correlated movement of ions with opposite charges hampers the overall ionic conductivity by decreasing the net charge transport. Figure 2 illustrates the contributions due to self- and cross correlations of the cations and anions. It can be observed that contributions due to self-correlation of both cations and anions are positive. Contribution due to self-correlations peaks at about *x*_IL_ = 0.3 for the anion (orange) and *x*_IL_ = 0.4 for the cation (blue). The self-correlation steadily decreases on either side of the peak ionic conductivity concentration. The decrease in the ionic conductivity due to self-correlation in the solvent-rich domain is related to a progressive reduction in the density of charge carriers while a drop in the ionic liquid-rich region can be explained by arrested motion of ions due to increasing ion–ion interactions in the ionic liquid. These steadily decrease with further increase in the ionic liquid concentration due to the increased ion–ion interactions.

Contributions to ionic conductivity due to cross-correlations may be resolved into three subcategories: cation–cation, anion–anion, and cation–anion interactions. These are presented in Figure 2 as purple, red, and green curves, respectively. Contribution due to cation–cation and anion–anion correlations was observed to be negative throughout the concentration range, while that due to anion–cation interaction was found to be positive. Details on the values are available in Table S5 in Supporting Information. The Nernst–Einstein conductivity arises due to contributions from self-cation and self-anion interactions, while the Einstein formalism estimates ionic conductivity by as the net summation of contributions due to self-correlation and cross-correlation terms from each ion. Optimum conductivity is obtained where the net summation of self-correlation terms and cation–anion cross correlation terms is maximum, near *x*_IL_ = 0.4. These observations are comparable with those reported by McDaniel and Son^26^ for [BMIM][BF_4_] in polar solvents such as acetonitrile and water. While this approach helps to resolve the ionic conductivity into its components, it is tedious and comes at an additional computational cost that may not be practicable for high-throughput screening applications. Therefore, below, we present a physically motivated and molecular-based approach taking into account fluctuations of ions in the first solvation shell of cations and anions.

### Cluster Analysis

**Figure 3:**
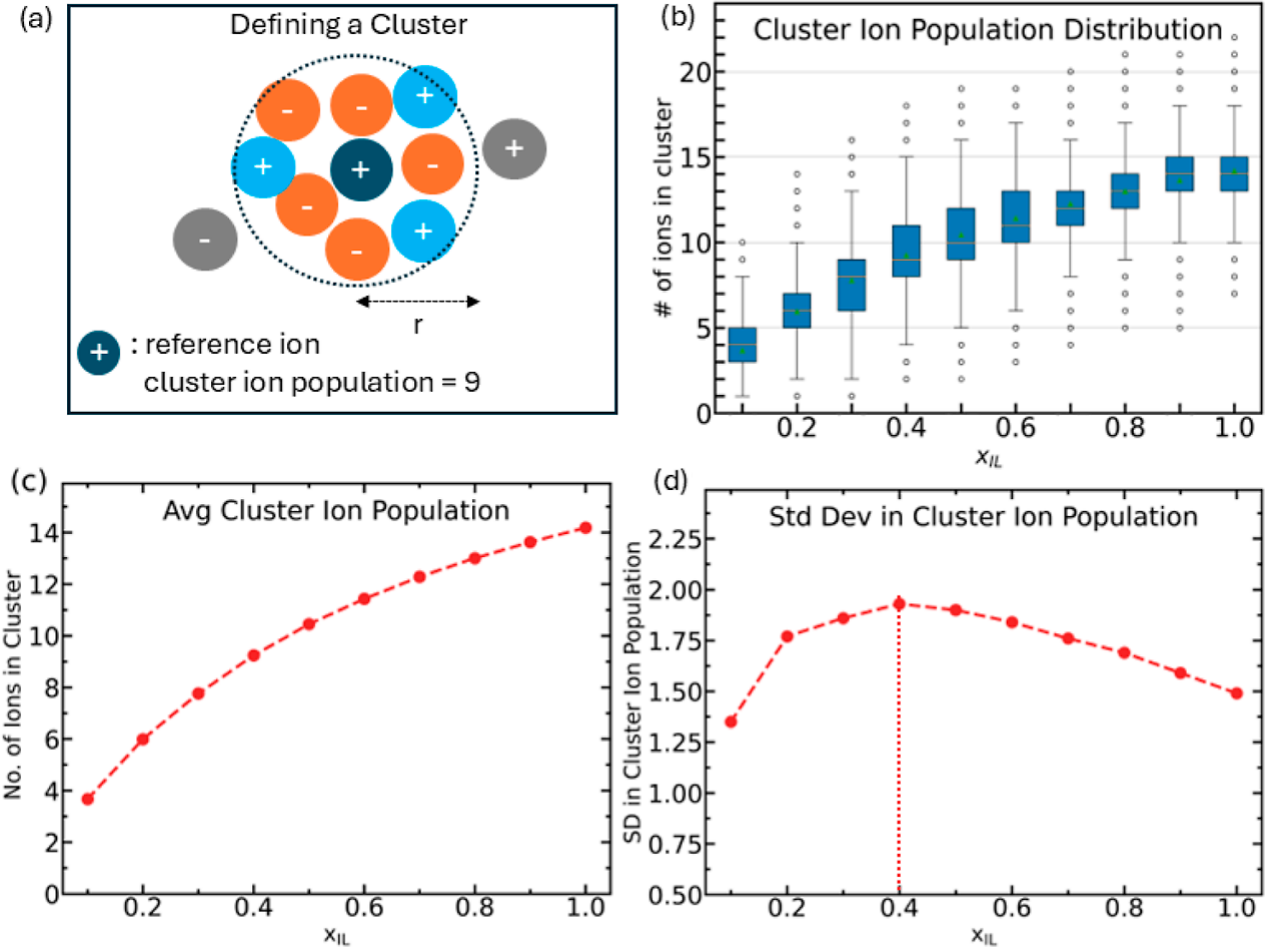
(a) Cluster is a group of both cations (blue) and anions (orange) located within a distance *r* from the reference ion. (b) Distribution of the total number of ions in a cluster. The dots represent outliers in the cluster populations representing transient states that occur rarely. (c) Average number of ions in a cluster. (d) Standard deviation (SD) in the cluster ion population. The vertical dotted line represents the mole fraction with the largest SD in the cluster ion population.

Figure 3a illustrates the definition of a cluster. A spherical region of radius 7.5 Å, equal to the radius of the first solvation shell from cation–anion RDF (Figure S3c in Supporting Information), was scanned around the center of mass of each ion to detect other ions. This group of ions was denoted as the cluster around the specific reference ion. Cluster analysis was performed for each ion using 1000 frames taken 20 ps apart. Cluster ion population was defined as the total number of cations and anions present within the spherical region. The ion–ion, ion–solvent, and solvent–solvent interactions in the mixtures make the systems dynamic at the molecular level. Therefore, continuous movement of ions and solvent molecules leads to a broad range of local compositions in clusters, giving rise to nonuniform distribution of ions. Figure 3b illustrates the cluster ion population distribution as it evolves with the ionic liquid concentration. The ion population distributions at each *x*_IL_ are given in detail in Figure S4 in Supporting Information. The cluster ion population has a spread of about four ions around the mean number of ions, meaning any clusters with ion population deviating beyond four ions more or less than the mean are rare and account for less than one percent of the total samples. It can be observed that completely dissociated ions, i.e., ions without any other ions within the first solvation shell can be observed only up to *x*_IL_ = 0.3 but are absent at higher concentrations. Ion-pairs, on the other hand, with exactly two ions within a solvation shell, are observable in systems up to *x*_IL_ = 0.5. The overall proportion of highly dissociated ions and ion-pairs is relatively low and decreases with ionic liquid concentration.

Figure 3c shows the mean ion population of the clusters. The average number of ions within a cluster increases with ionic liquid concentration, which can be explained on the basis of an increase in the number density of ions. The cluster ion population in neat [EMIM][BF_4_] is approximately 3.5 times that at *x*_IL_ = 0.1. In order to determine changes in the cluster population, the standard deviation is computed and plotted in Figure 3d as a function of ionic liquid concentration. Variation in the cluster ion population may be interpreted as an indicator of how tightly ions are held together. Frequent movement of ions relative to each other leads to a wider spread of cluster ion population. Such exchanges will also contribute toward rapid charge transport and consequently yield higher ionic conductivity. This is evident from the mole fraction with the highest standard deviation in the cluster ion population at *x*_IL_ = 0.4; it is worth noting that, experimentally, a maximum in the ionic conductivity is found close to this ionic liquid concentration.

In dilute systems, ions travel faster and longer distances before encountering other ions; hence, the overall population variance within a cluster is low. In concentrated systems, the motion of an ion is hindered by other ions in its vicinity, and the crowding of ions prevents drastic changes in the immediate environment of an ion, thereby yielding low population variation. However, at the optimum composition, a balance between the overall number of ions and individual diffusion rates leads to a very dynamic solvation environment, producing a larger variation in the solvation shell of an ion. Thus, population variation can be used to qualitatively estimate the extent of dynamism within a system.

### Cage Correlations

**Figure 4:**
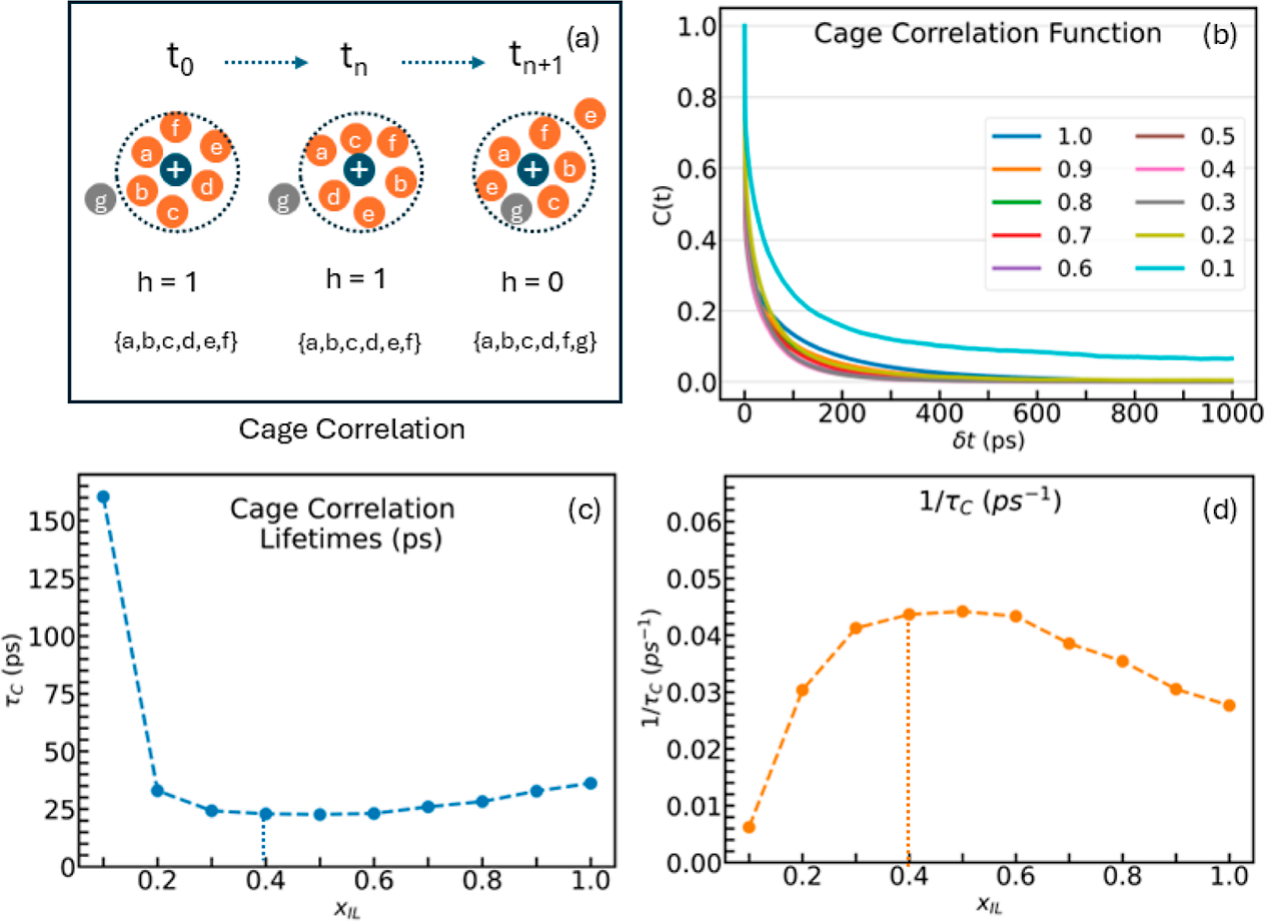
(a) A cage is identified by the set of oppositely charged ions around the reference ion. (b) Cage correlation functions. (c) Cage correlation lifetime τ_C_. (d) Inverse of cage correlation lifetimes 1/τ_C_. Vertical dotted lines represent the optimum values.

Cluster analysis provides insight into the ion demographics. However, it lacks quantitative metrics of the movement of ions and dynamics in and out of the solvation shell surrounding the ions. The dynamic aspects of the solvation environment can be captured using a cage correlation analysis. An illustration of a cage around an ion is provided in Figure 4. A spherical region of radius 7.5 Å, equal to the radius of the first solvation shell from the cation–anion RDF, was scanned around the center of mass of each ion to detect ions carrying charge opposite to that of the reference ion. For cations, this constitutes the anions in the first solvation shell, while for anions, this is the group of cations within the first solvation shell. To quantify the dynamics of oppositely charged ions in these solvation shells, autocorrelation of a Heaviside function was calculated for a given time *t*.
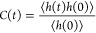
10

In this equation, *h*(*t*) is a Heaviside function, which takes the value of 1 if the identity of the ions in a solvation shell is continually maintained for the entirety of the time *t*; otherwise, the Heaviside function takes the value of 0. Figure 4a depicts a cage formed by a set of anions labeled {*a*,*b*,*c*,*d*,*e*,*f*} at *t* = 0 around a cation. At time *t* = *t*_*n*_, the cation and the surrounding anions may have moved to different location; however, the solvation shell around the cation is still occupied by the same anions as *t* = *t*_0_; therefore, *h*(*t*_*n*_) = 1 for this cation. On the other hand, at time *t*_*n*+1_, one of the anions has crossed the boundary of the solvation shell and another anion has entered the solvation shell. As the original identity of the anions has not been maintained, *h*(*t*_*n*+1_) = 0. The average value of correlation function *C*(*t*) is calculated as an ensemble average of the Heaviside function for all ions at a given time, as shown in eq 10. For the calculation of cage correlation function, each ion was tracked by assigning a unique identity. Cage correlation analysis was performed on all ions over 1000 snapshots spaced 1 ps apart using the time-origin shifting method for better statistical accuracy.

Figure 4b shows the correlation functions for different ionic liquid concentrations. It was observed that the correlation functions exhibit a nonmonotonic behavior with the ionic liquid concentration. The slowest decay in the correlation function was observed in the most dilute system with *x*_IL_ = 0.1, which was followed by the neat ionic liquid. These observations are in agreement with theoretical expectations: the correlation function for a dilute system decays to zero more slowly as compared to that of other systems because single, dissociated ions shielded by solvent or associated ion-pairs form stable solvation environments and maintain the cage identity for much longer as compared to more populated cages. On the other hand, a neat ionic liquid is saturated with ions, and hence, the cages are densely packed. Strong ion–ion interactions and a large population of ions hinder the rapid movement of ions in and out of the cage. This results in more strongly correlated ion cages and hence a slowly decaying correlation function. Unlike the dilute system, the correlation function in neat ionic liquid drops to zero faster due to the larger ion population as well as the lack of dissociated ions or ion-pairs shielded by the solvent.

Cage correlation lifetimes were estimated by fitting the correlation function values to eq 12 and can be integrated analytically by using eq 14.
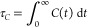
11
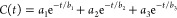
12
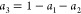
13
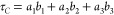
14

Figure 4c displays the average cage correlation lifetimes τ_C_ at various ionic liquid concentrations. It can be observed that the correlation lifetime at the lowest concentration (*x*_IL_ = 0.1) is about 160 ps, which is roughly four and six times the correlation lifetimes at other concentrations. The correlation lifetimes pass through a minimum at *x*_IL_ = 0.4, the concentration corresponding to the peak in the ionic conductivity. The differences in the cage correlation lifetimes are subtle compared with the correlation time at the lowest dilution. A clearer picture emerges, when the inverse of the cage correlation lifetimes is plotted against the ionic liquid concentrations, as shown in Figure 4d. The ionic liquid concentration at which a maximum in inverse correlation lifetime is obtained nearly coincides with the concentration exhibiting a peak ionic conductivity.

Zhang and Maginn^38^ have demonstrated an inverse relationship between cage correlation lifetimes and self-diffusion coefficients in neat ionic liquids. In a binary mixture of an ionic liquid and molecular solvent, however, the self-diffusion coefficients are a monotonic function of ionic liquid concentration (Figure S2) while the cage correlation lifetimes pass through a minimum. Hence, such an inverse relationship between the cage correlation times and self-diffusion coefficients is absent in binary mixtures. The cage correlation lifetimes quantify the level of dynamism in a system and can be used as a guide toward determining the optimum ionic liquid mole fraction that yields peak conductivity. From a molecular perspective, the average lifetime a given set of oppositely charged ions is maintained can be linked to the strength of the ion–ion interactions. Weak ion–ion interactions and consequently faster cage dynamics lead to a shorter correlation lifetime, while a longer correlation time indicates stronger ion–ion interactions.

### Effect of Force Fields

Force fields are known to significantly influence the estimation of thermophysical properties along with the structure, distribution, and orientation of the chemical species within the system. While researchers such as Avula et al.^41^ have proposed elaborate workflows for force field optimization and accurate prediction of ionic conductivity, implementing such workflows is challenging for high-throughput screening applications. Force field optimization requires reliable experimental data, the availability of which would render much of the computational effort redundant. Furthermore, force fields are often optimized for pure ionic liquids and may not be suitable for use in binary mixtures with molecular solvents due to the polarization effects. Hence, researchers are compelled to rely on the results using generic, unoptimized force fields for preliminary investigations. To verify whether the methods developed in this work are applicable across force fields, we tested them using three different force fields used to represent [EMIM][BF_4_] in EG. Among these, the VSIL^54^ and 0.8*2009IL^57,58^ force fields impose a net charge of 0.8 and −0.8 on the cations and anions, respectively, with the charges remaining fixed through the different mixture compositions. Both the force fields, thus, do not take into account charge polarization effects due to the interaction with the molecular solvent. We also tested effects of polarization due to the molecular solvent by updating the atomistic charges using the DFT-based density derived atomistic potential^59^ (DDAP) approach at every composition.^59^ The net charge on the ions in this case varies as a function of the ionic liquid mole fraction in the mixture. The magnitude of charges on cation and anion are slightly different due to polarization as small charge (typically less than 0.05e) is induced on the solvent molecules. (Detailed charges on ions are provided in Table S10 in Supporting Information.) It is important to note that the contribution of solvent molecules toward ionic conductivity is negligible as conductivity scales per square of the net charge and is thus insignificant at very low net charge.

**Figure 5:**
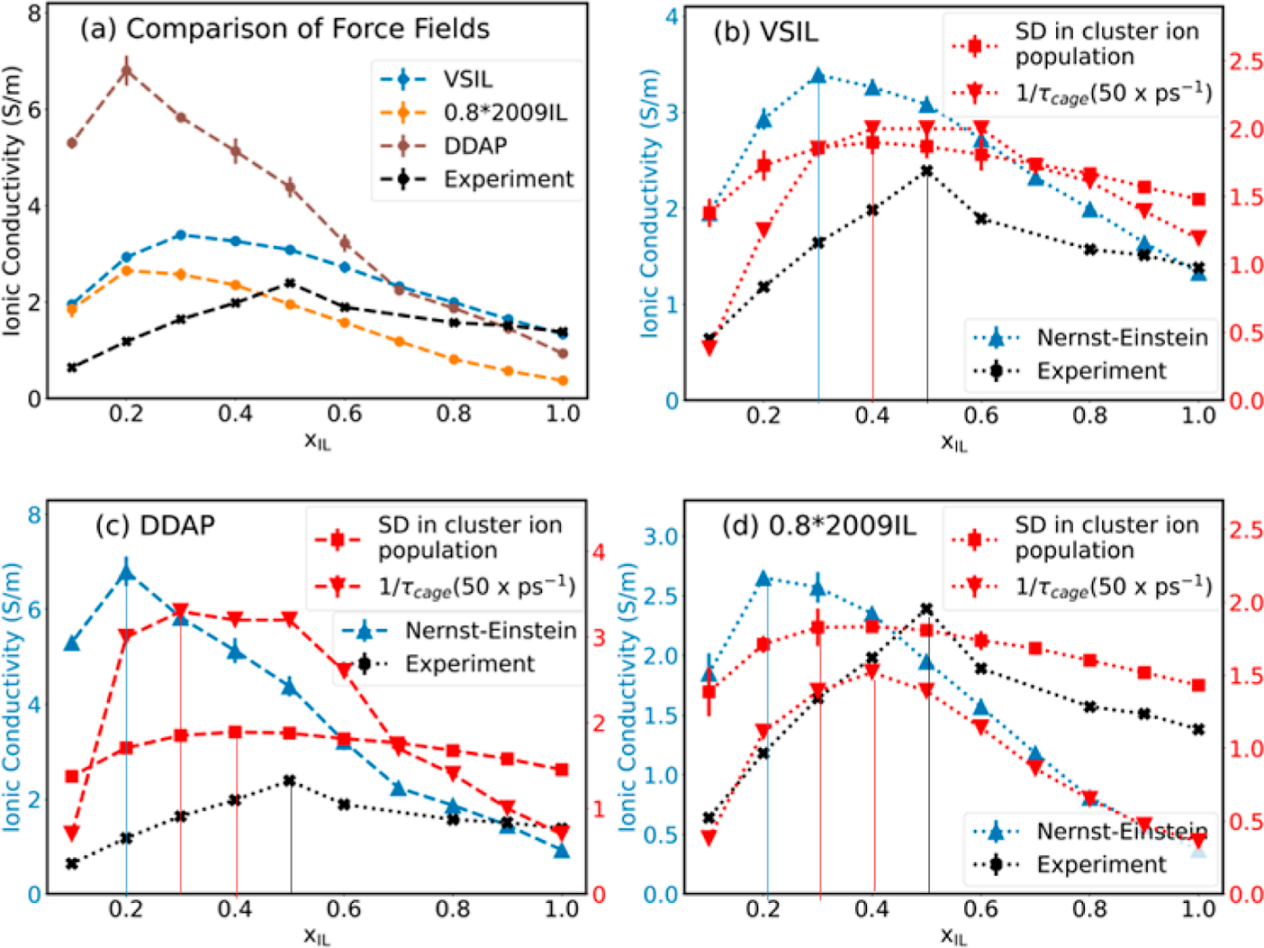
(a) Nernst–Einstein conductivity predicted using different force fields. Primary axis: Nernst–Einstein (blue) and experimental (black) conductivity values; secondary axis: SD in cluster ion population (red squares) and 1/τ_cage_ values scaled by 50 (red triangles) for ease of representation using (b) VSIL, (c) DDAP, and (d) 0.8 × 2009IL force fields. Vertical lines indicate corresponding optimum values.

Figure 5a depicts the ionic conductivity values calculated using the Nernst–Einstein formalism for the three force fields. The plot also shows the experimental values for comparison. It may be observed that all force fields capture the nonmonotonous trend in ionic conductivity albeit differences in the actual values or the compositions with peak Nernst–Einstein conductivity. These observations are in agreement with theory given that diffusion-based Nernst–Einstein estimates ignore ion–ion interactions and that the force fields are not optimized for the estimation of transport properties. Figures 5b–d represents the analyses from the individual force fields VSIL, DDAP, and 0.8*2009IL, respectively. The Nernst–Einstein and experimental ionic conductivity values are shown on the primary axis. The standard deviation in the cluster ion population and the inverse of the cage correlation lifetime (1/τ_C_) are shown on the secondary axis (red). 1/τ_C_ is scaled by a factor of 50 for ease of representation using the same plot. In all three cases, we observe the optimum mole fraction predicted based on SD in cluster ion population and inverse of cage correlation lifetimes is closer to optimum mole fractions obtained from experiment as compared to the Nernst–Einstein prediction.

### *NVT* vs *NPT* Ensemble

To test whether the approach is independent of the choice of the ensemble, we first demonstrated that the behavior is reproduced in the *NPT* ensemble as well, so that an additional calculation in the *NVT* ensemble is not required. Table S7 from Supporting Information compares the ionic conductivity values and variation in cluster-ion population obtained using the *NPT* ensemble with those obtained from the *NVT* ensemble for [EMIM][BF_4_] and EG at 298 K. It is observed that both ensembles result in similar ionic conductivity values and overall trend in fluctuations within the local environment. This enables us to make a qualitative determination of high ionic conductivity mixing ratios for binary mixtures of ionic liquids and molecular solvents. Next, we tested our approach on several ionic liquid-molecular solvent electrolytes with experimental conductivity values available in literature.

### Other Systems

#### Effect of Anions

**Figure 6:**
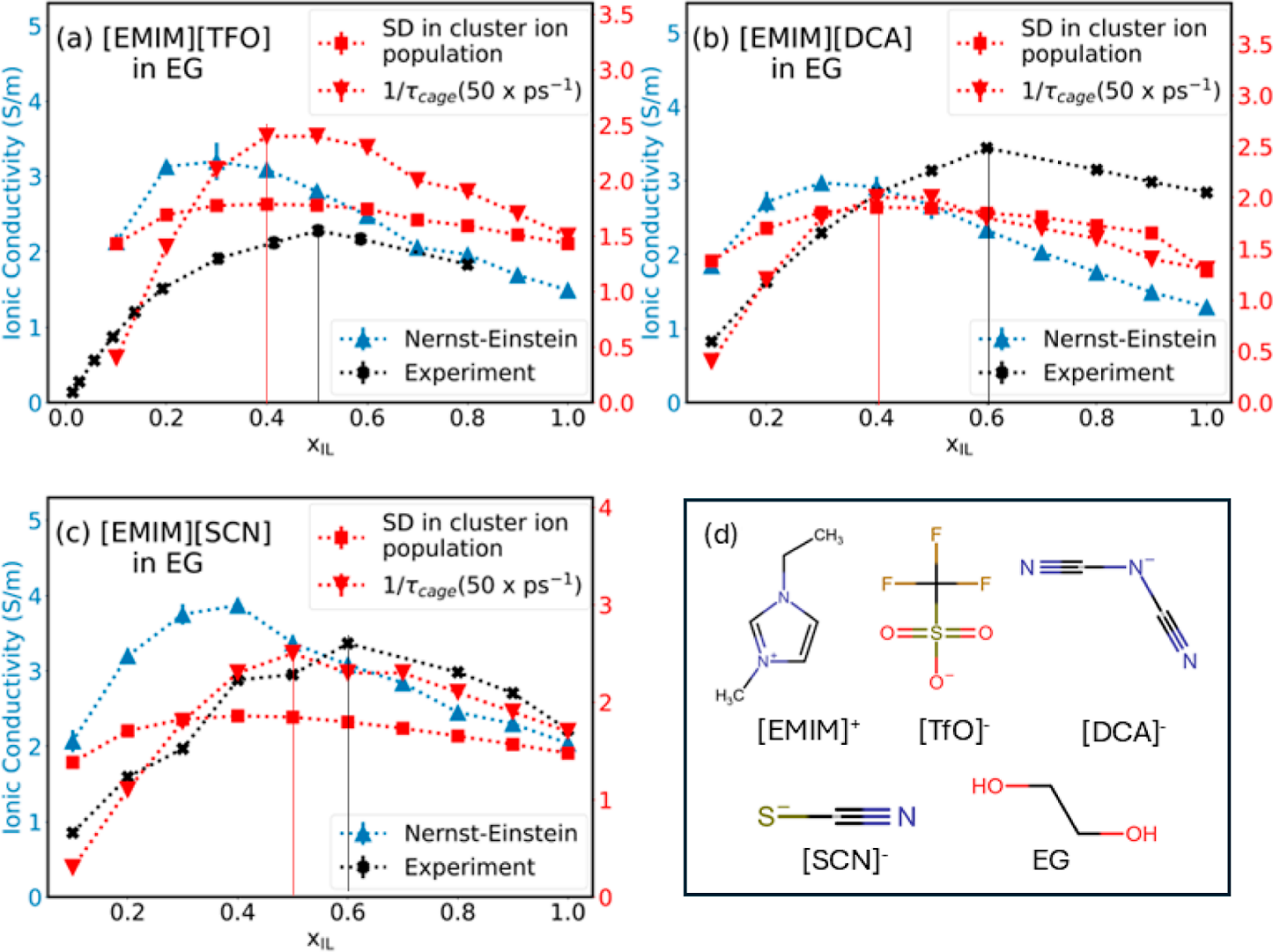
Primary axis: Nernst–Einstein (blue) and experimental (black) conductivity values; secondary axis: SD in cluster ion population (red squares) and 1/τ_cage_ values scaled by 50 (red triangles) in electrolytes with EG and ionic liquids (a) [EMIM][TfO] (at 313 K), (b) [EMIM][DCA], and (c) [EMIM][SCN] and (d) chemical structures of constituents.

Figure 6a–c represents systems containing binary mixtures of ionic liquids containing [TfO]^−^, [DCA]^−^, and [SCN]^−^, respectively. These anions represent a variety of fluorinated and nonfluorinated species with varying affinity toward CO_2_. These are of interest for the MAMG^50^ process for electrochemical CO_2_ capture mentioned earlier. The results for [EMIM][TfO] in EG were compared with the values reported by Nordness et al.,^76^ while those for [EMIM][DCA] and [EMIM][SCN] were compared with the values reported by Chauhan et al.^19^ It was observed that the mole fractions associated with peak conductivity obtained from the analyses of features of the solvation environment matched closely with the experimental measurements reported in the literature. Detailed values for each system are available in Supporting Information (Tables S11-S13).

We observed that the most dynamic systems based on the analysis of cage correlation lifetimes occur at *x*_IL_ = 0.4, while the standard deviation in cluster ion population peaks at *x*_IL_ = 0.3. These differences are likely attributed to the differences between the center of mass and center of charge for these anions as the solvation shell was defined based on the center of mass. Regardless, these compositions are better predictors of the optimum compositions for peak conductivity as compared to those predicted using the Nernst–Einstein approach.

#### Effect of Cations and Solvents

**Figure 7:**
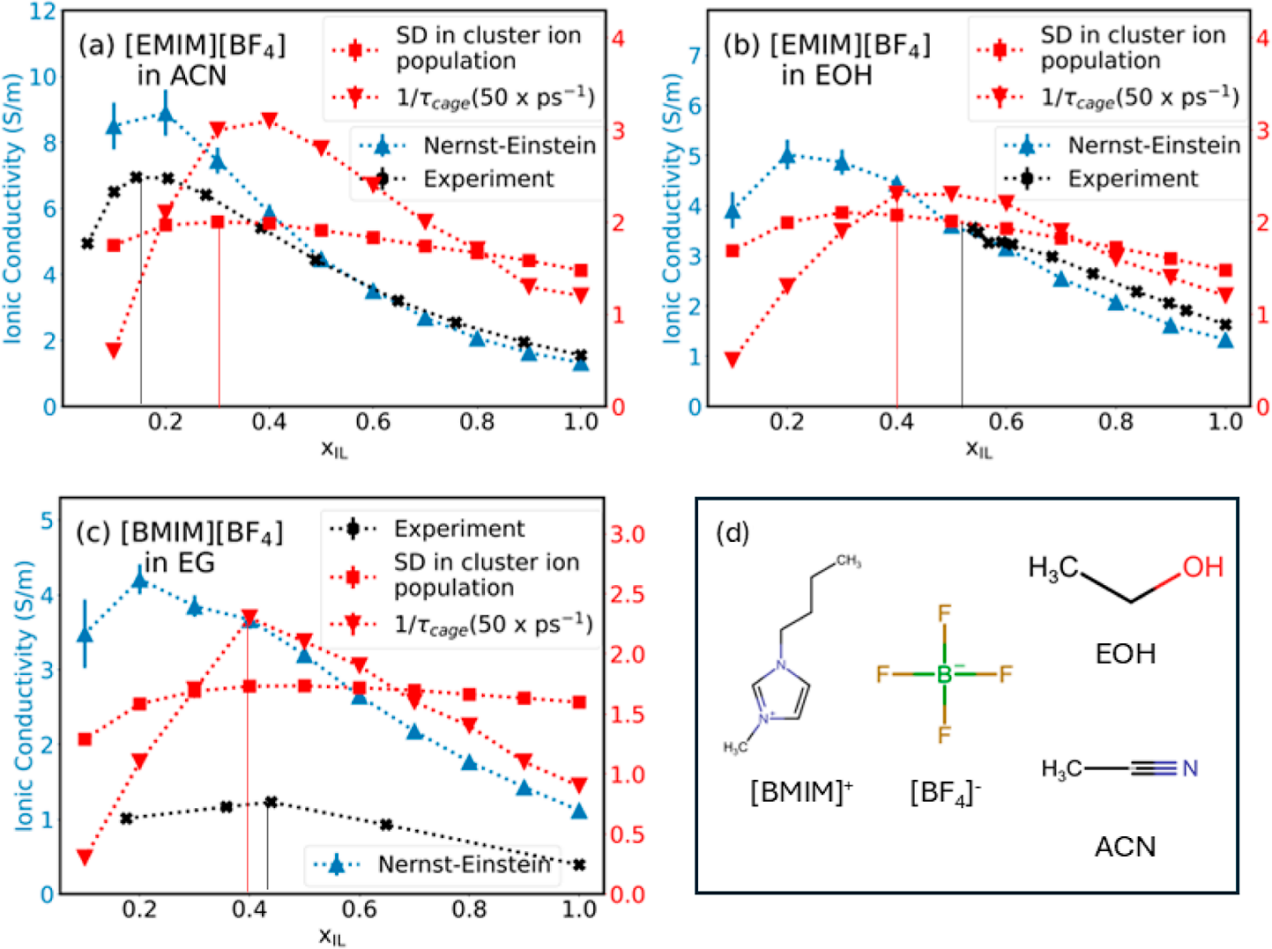
Primary axis: ionic conductivity values: Nernst–Einstein (blue) and experiment (black); secondary axis: SD in the cluster ion population (red squares) and 1/τ_cage_ values scaled by 50 (red triangles) in electrolytes (a) [EMIM][BF_4_] in ACN, (b) [EMIM][BF_4_] in EOH, and (c) [BMIM][BF_4_] in EG (333 K) and (d) chemical structures of constituents.

Figure 7a,b illustrates the behavior of [EMIM][BF_4_] in ACN and EOH, respectively, which represent solvents with different size, polarity, hydrogen bonding capability, and dielectric strength. Figure 7c represents mixtures of [BMIM][BF_4_] in EG to test the proposed approach for systems containing other cations. The experimental values for [BMIM][BF_4_] in EG were taken from work by Croitoru et al.,^77^ while those for [EMIM][BF_4_] in ACN and EOH were taken from the works by Stoppa et al.^23^ and Rilo et al.,^78^ respectively. We observed that the trend in the standard deviation in the cluster ion population dampens slightly in the case of [BMIM][BF_4_], perhaps due to additional flexibility offered by the longer chain length. However, the 1/τ_C_ continues to exhibit a pronounced nonmonotonous behavior, which can help identify compositions with peak ionic conductivity.

We observe good agreement between the optimum compositions predicted using dynamics of the solvation environment and those from experiments for [BMIM][BF_4_] in EG and [EMIM][BF_4_] in EOH systems. On the other hand, Figure 7a illustrates that for the [EMIM][BF_4_] in the ACN system, the *x*_IL_ with peak Nernst–Einstein conductivity is closer to the optimum composition as observed from experiment. This may be attributed to the high polarity and smaller size of the acetonitrile molecule, which leads to stronger shielding of the ions from each other and thus a higher degree of solvation. In effect, it reduces the ion–ion interactions, in line with the assumptions in the Nernst–Einstein approach.

Remarkably, despite the differences in the chemical structure of anions (fluorinated vs nonfluorinated) or cations, the most dynamic solvation environments indicated by maximum variability in the ion population in the first solvation shell are observed in mixtures at similar compositions to those which yield the peak ionic conductivity. Furthermore, systems containing solvents of varying polarity (Figure 7a,b) also exhibit similar behavior. This suggests that the trend in the bulk ionic conductivity in electrolytes may be rooted at the molecular level in the fluctuation of the ion population around ions. This is further evidenced by the nonmonotonous trend in the τ_C_ values which pass through a minimum at mole fractions that are closer to mole fractions that exhibit peak ionic conductivity.

## Conclusions

This work investigated the trends in the ionic conductivity of a binary mixture of [EMIM][BF_4_] and EG at various mole fractions between 0.1 and 1.0 at 298 K and 1.0 bar using MD simulations. The predictions obtained using Nernst–Einstein and Einstein formalisms were compared with the experimentally measured values. Both approaches successfully predict the nonmonotonous ionic conductivity as a function of the ionic liquid concentration. However, the predicted peak conductivity occurs at *x*_IL_ = 0.3 while the measured peak conductivity occurs at *x*_IL_ = 0.5; the difference could be attributed to the fact that force field parameters are not optimized for the prediction of ionic conductivity. Due to the rigorous approach of considering all ion–ion interactions, the predictions obtained using the Einstein approach match well with the experimental values. However, these calculations require long trajectories of approximately 100 ns for sufficient sampling, which makes the use of this approach computationally expensive for large-scale screening. Resolution of the ion–ion interactions into self- and cross-correlation components sheds light on the contribution due to cation–cation, cation–anion, and anion–anion interactions. It was observed that ionic conductivity was predominantly driven by the self-correlation terms and aided by cation–anion cross correlations, while the cation–cation and anion–anion cross correlations hinder the overall ionic conductivity.

The second part of this work focused on a molecular-level explanation of trends in ionic conductivity with the changes in the solvation environment of ions as a function of the ionic liquid concentration, which was quantified in terms of the total number of ions around an ion in the first solvation shell and the corresponding fluctuations. Surprisingly, this approach revealed that the fluctuations in the ion population showed a nonmonotonous trend with the concentration. More importantly, the ionic liquid concentration corresponding to the maximum variability in the fluctuations was found to be in close agreement with the ionic liquid concentration at which the maximum ionic conductivity was observed. Investigation of systems containing various ionic liquids with EG and [EMIM][BF_4_] with solvents of varying polarity showed that the connection between fluctuation in the local ion population is likely to be a universal predictor of the concentration at which the peak ionic conductivity in ionic liquid-solvent can be obtained. It was also demonstrated that the inverse of the cage correlation lifetimes could also be a viable predictor of the optimum mixture composition.

This work illustrates that while rigorous approaches such as the Einstein formalism can be leveraged to predict ionic conductivity values, investigation of the first solvation shell through cluster analysis and cage correlation can provide valuable guidance in identifying mole fractions with peak ionic conductivity in ionic liquid–solvent mixtures. Cluster analysis and cage correlation lifetimes require a short trajectory, as short as 1–2 ns in comparison to 100 ns long trajectories required for computing ionic conductivity using Einstein formalism. Hence, investigation of the first solvation shell can be used in tandem with the traditional Nernst–Einstein and Einstein approaches for efficiently screening binary mixtures of ionic liquids and molecular solvents and determining optimum mixing ratios.

## Supporting Information Available

Detailed results: density, excess molar volume, self-diffusion coefficients, ion–ion correlations, RDFs, comparison of Nernst–Einstein and Einstein conductivity, *NVT* vs *NPT* ensembles, and cluster analysis; and graphical summary and results for [EMIM][BF_4_] in EG (VSIL), [EMIM][BF_4_] in EG (0.8 × 2009IL), [EMIM][BF_4_] in EG (DDAP), [EMIM][TfO] in EG, [EMIM][DCA] in EG, [EMIM][SCN] in EG, [EMIM][BF_4_] in ACN, [EMIM][BF_4_] in EOH, and [BMIM][BF_4_] in EG (PDF). Sample input files and analysis scripts are available from https://github.com/ShahResearchGroup/JCTC_2025.
