# Supplementary material for: Identifying High Ionic Conductivity Compositions of Ionic Liquid Electrolytes Using Features of the Solvation Environment
Source: J Chem Theory Comput. 2025 Feb 11;21(4):1929–40. doi: 10.1021/acs.jctc.4c01441 (PMC11866759; doi:10.1021/acs.jctc.4c01441)
Supplement: Supplementary file 1 — ct4c01441_si_001.pdf [file ct4c01441_si_001.pdf]

Supporting Information
Identifying High Ionic Conductivity Compositions of Ionic Liquid Electrolytes Using Features of the Solvation Environment

Amey Thorat,† Ashutosh Kumar Verma,† Rohit Chauhan,‡ Rohan Sartape,‡ Meenesh R. Singh,‡ and Jindal K. Shah*,†

†*School of Chemical Engineering, Oklahoma State University, Stillwater, Oklahoma 74078, United States*

‡*Department of Chemical Engineering, University of Illinois at Chicago, Chicago, Illinois 60608, United States*

E-mail: jindal.shah@okstate.edu

Contents

S1 Abbreviations and List of Force Fields S3

S2 $[EMIM][BF_4]$ in EG S4

S2.1 VSIL S4

S2.1.1 Density and Excess Molar Volume S4

S2.1.2 Self-Diffusion Coefficients S5

S2.1.3 Ion-Ion Correlations S7

S2.1.4 Radial Distribution Functions S8

S2.1.5 Comparison of Nernst-Einstein vs. Einstein Conductivity S10

S2.1.6 Ionic Conductivity Estimation using NPT and NVT Ensembles . . . S10

S2.1.7 Cluster Analysis S11

S2.1.8 Summary of Results S12

S2.2 0.8*2009IL S14

S2.3 DDAP S16

S3 Other Systems S18

S3.1 [EMIM][TfO] in EG S18

S3.2 [EMIM][DCA] in EG S20

S3.3 [EMIM][SCN] in EG S22

S3.4 $[EMIM][BF_4]$ in ACN S24

S3.5 $[EMIM][BF_4]$ in EOH S26

S3.6 $[BMIM][BF_4]$ in EG S28

S1 Abbreviations and List of Force Fields

Table S1: List of Abbreviations

Avg	Average
SD	Standard Deviation
x_{IL}	Mole fraction of ionic liquid (simulation)
$x_{IL_{exp}}$	Mole fraction of ionic liquid (experiment)
q_+	Net charge on cation
q_-	Net charge on anion
IC_{NE}	Nernst-Einstein conductivity (S/m)
IC_{EN}	Einstein conductivity (S/m)
IC_{exp}	Experimentally measured conductivity (S/m)
CIP	Cluster ion population
τ_C	Cage correlation lifetime

Table S2: List of Force Fields

Species	Abbreviation	Force Field
Cations		
1-butyl-3-methylimidazolium	BMIM	VSIL[1]
1-ethyl-3-methylimidazolium	EMIM	VSIL[1]
1-ethyl-3-methylimidazolium	EMIM	0.8*2009IL[2,3]
1-ethyl-3-methylimidazolium	EMIM	OPLS with DDAP[4] derived atomistic charges
Anions		
dicyanamide	DCA	VSIL[1]
tetrafluoroborate	BF_4	VSIL[1]
thiocyanate	SCN	VSIL[1]
triflate	TfO	VSIL[1]
Solvents		
acetonitrile	ACN	OPLS (LigParGen)[5–7]
ethanol	EOH	OPLS (LigParGen)[5–7]
ethylene glycol	EG	OPLS-DES[8]

S2 [EMIM][BF_4] in EG

S2.1 VSIL

S2.1.1 Density and Excess Molar Volume

Accurate prediction of density serves to screen out any unphysical results from simulations. Excessive deviations from the experimental values may indicate errors in simulation, or unsuitable force fields. In agreement with the values reported by Doherty et al. for pure [EMIM][BF_4], the predicted density using VSIL force field matches well with experimental values. For pure EG, the OPLS_DES force field yields density within 3% of the experimental density. Figure S1 illustrates a slight increase in the overall density with the addition of [EMIM][BF_4]. Excess molar volume also increases with ionic liquid concentration. Strong EG-[EMIM][BF_4] interaction is likely to produce negative deviation in the excess molar volumes in the solvent-rich systems, while the slightly positive excess molar volumes in the ionic liquid rich systems are likely due to weaker electrostatic interactions among the ions, considering that the ionic charges are fixed at 0.8. Overall, the magnitudes of excess molar volumes are fairly low, upto 0.2 cc/mol.

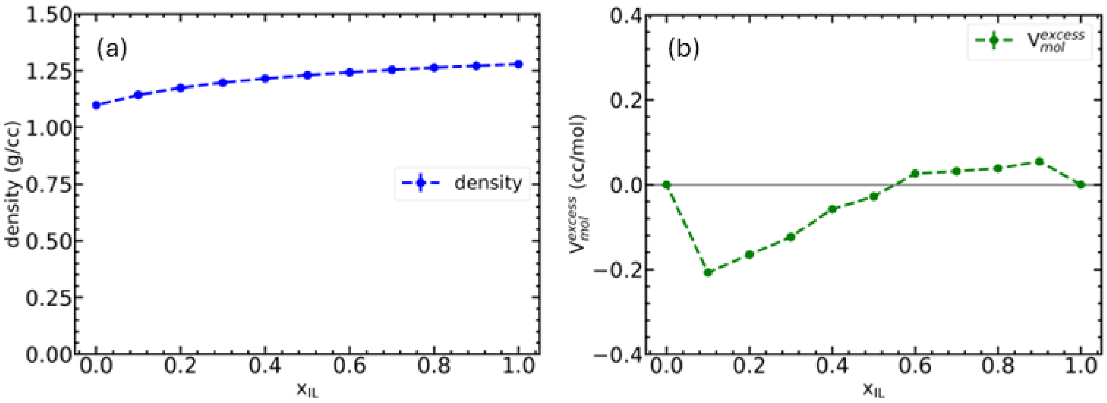

Figure S1: Predicted density and excess molar volume as function of [EMIM][BF_4] mole fraction in EG at 298 K

Table S3: Simulated Density and Excess Molar Volume for [EMIM][BF_4] in EG at 298 K

x_{IL}	Ion Pairs	Mass Density (g/cc)	Excess Molar Volume (cc/mol)	Molar Density (mmol/cc)
1.0	500	1.278	0.000	6.456
0.9	450	1.270	0.054	6.891
0.8	400	1.262	0.038	7.393
0.7	350	1.253	0.031	7.973
0.6	300	1.242	0.026	8.652
0.5	250	1.230	-0.028	9.461
0.4	200	1.214	-0.058	10.435
0.3	150	1.197	-0.124	11.636
0.2	100	1.173	-0.165	13.147
0.1	50	1.143	-0.207	15.108
0.0	0	1.097	0.000	

S2.1.2 Self-Diffusion Coefficients

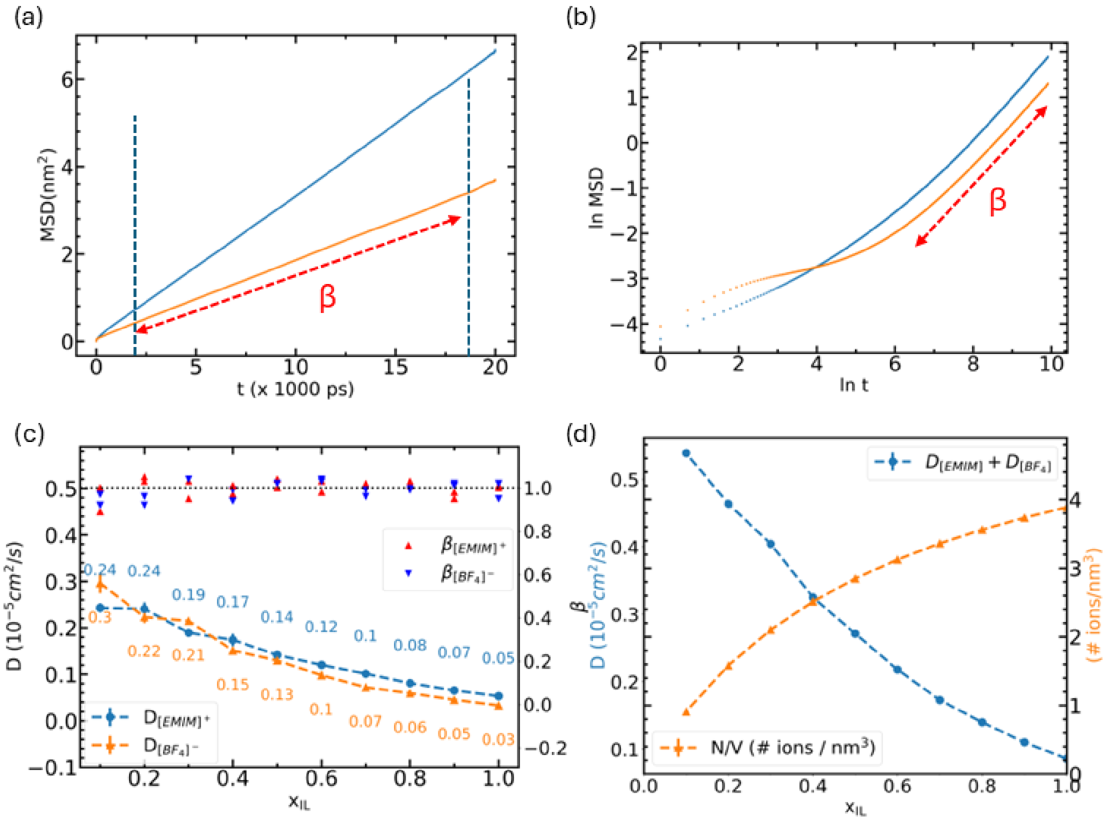

Figure S2: a) Mean squared displacement (MSD) as a function of time (t) b) ln MSD vs. ln t with beta values indicating slope in the linear region c) Self-diffusion coefficients for $[EMIM]^+$ and $[BF_4]^-$ and corresponding β values d) summation of self-diffusion coefficients and ionic density as a function of ionic liquid concentration

Figure S2a and Figure S2b represent the mean square displacement (MSD) for cations and anions as a function of simulation time and their log values respectively. Figure S2c illustrates the self-diffusion coefficients for $[EMIM]^+$ and $[BF_4]^-$ calculated as the slope of ln(MSD) vs. ln(t) where the MSD evolves linearly with time. The mean estimated self-diffusion coefficient for $[EMIM]^+$ in the neat ionic liquid is 0.053x10^{-5} cm^2s^{-1} while that for $[BF_4]^-$ is 0.033x10^{-5} cm^2s^{-1}. The reported values by Noda et al.[9] for self-diffusion coefficients in neat $[EMIM][BF_4]$ for cation and anion are slightly higher at 0.079x10^{-5} cm^2s^{-1} and 0.062x10^{-5} cm^2s^{-1} respectively. The difference between predicted and experimental values may be attributed to the force field effects and relatively low temperature of 298 K for estimation of diffusion coefficients. However, it can be observed that the trend is preserved and the cation tends to diffuse faster than the anion in the ionic liquid rich systems. Anion diffusion catches up with the cation diffusion and surpasses it in the dilute systems due to smaller size of anions as compared to the cation. Overall, the diffusion coefficients decrease with the addition of ionic liquid to the mixture. The diffusion coefficients for the cation and anion in the most dilute system with x_{IL}=0.1 are between 5 to 10 times the corresponding values in pure $[EMIM][BF_4]$. This increase may be attributed to the increased dissociation, reduced viscosity, and weaker ion-ion interactions as compared to pure ionic liquids. Exponential increase in the self-diffusion coefficients for cations and anions with dilution is observable in similar systems with polar solvents due to higher degree of dissociation than dilution with solvents of low dielectric strength such as dichloromethane.[10]

Table S4: Self-Diffusion Coefficients for $[EMIM]^+$ and $[BF_4]^-$ at 298 K (10^{-10} m^2/s)

x_{IL}	Avg $D_{[EMIM]^+}$	SD $D_{[EMIM]^+}$	Avg $D_{[BF_4]^-}$	SD $D_{[BF_4]^-}$
1.0	0.530	0.002	0.330	0.0008
0.9	0.653	0.003	0.452	0.0006
0.8	0.808	0.002	0.593	0.0033
0.7	1.011	0.003	0.719	0.0030
0.6	1.203	0.007	0.978	0.0080
0.5	1.416	0.006	1.299	0.0020
0.4	1.739	0.013	1.512	0.0070
0.3	1.896	0.004	2.148	0.0015
0.2	2.406	0.014	2.232	0.0131
0.1	2.430	0.010	2.961	0.0214

S2.1.3 Ion-Ion Correlations

Table S5: Contribution to Ionic Conductivity (S/m) from Ion-Ion Correlations for $[EMIM][BF_4]$ in EG at 298 K

x_{IL}	σ^{self}_{cat}	σ^{self}_{ann}	$\sigma^{cross}_{cat-cat}$	$\sigma^{cross}_{ann-ann}$	$\sigma^{cross}_{cat-ann}$
1.0	0.84	0.52	-0.591	-0.118	0.634
0.9	0.99	0.66	-0.656	-0.132	0.752
0.8	1.13	0.82	-0.783	-0.329	0.617
0.7	1.29	1.00	-0.812	-0.311	0.743
0.6	1.45	1.20	-0.841	-0.410	0.763
0.5	1.59	1.40	-0.930	-0.493	0.651
0.4	1.68	1.59	-0.883	-0.625	0.675
0.3	1.65	1.66	-0.870	-0.494	0.539
0.2	1.45	1.57	-0.610	-0.494	0.219
0.1	0.93	1.10	-0.141	-0.181	0.014

S2.1.4 Radial Distribution Functions

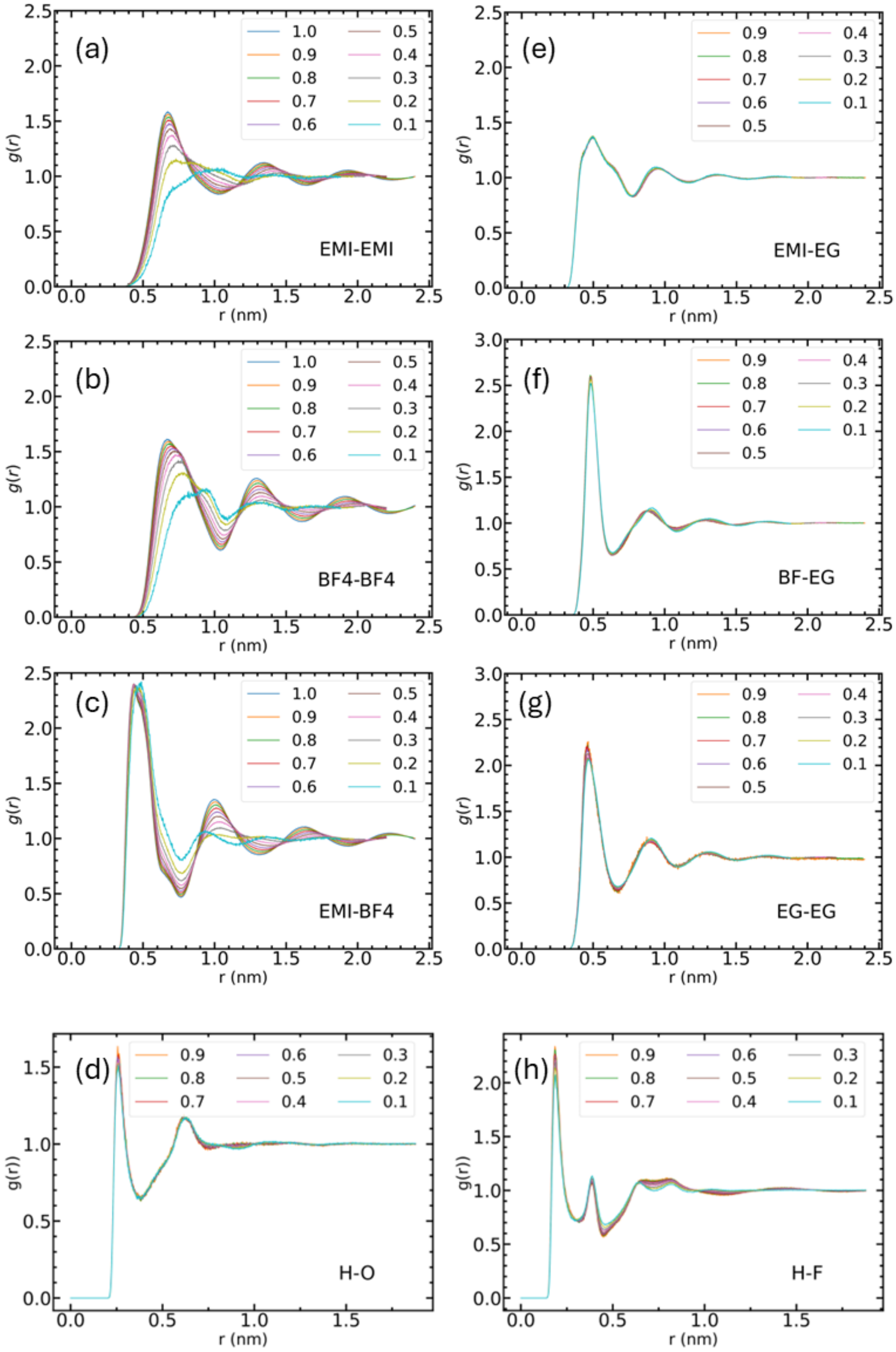

Figure S3: Radial distribution functions (RDFs) in [EMIM][BF_4] and EG (VSIL) a) cation-cation b) anion-anion c) cation-anion d) RDF between acidic H on imidazolium ring and O atoms of OH group on EG e) cation-solvent f) anion-solvent g) solvent-solvent h) RDF between F atom of BF_4 and H atoms on OH group in EG

Radial Distribution Functions (RDFs) estimate $g(r)$ as the ratio of local density to the bulk density of a species within a sphere of radius r around a reference. RDFs were calculated to gain insights into the solvation environment at different concentrations. Figure S3 illustrates various RDFs for the cation and anion. RDFs around atoms that can form hydrogen bonds have also been included to assess the degree of hydrogen bonding between anion-solvent, and solvent-solvent pairs. Figure S3a and S3b depict the cation-cation and anion-anion RDFs respectively. For cation-cation and anion-anion RDFs, the height of the first peak decreases with dilution because ions with like charges remain to stay as far from each other to minimize repulsion. This is also reflected in the inverse relation between the radii of first solvation shells (first minima) and ionic liquid concentration. Dilute systems have larger solvation shell radii for ions with like charges. On the other hand, almost equal $g(r)$ values for the first peak in the cation-anion RDF Figure S3c indicate that the local density of oppositely charged ions tends to increase proportionately with the bulk density. This is expected due to charge neutrality. The first solvation shell radii for oppositely charged ions remains almost constant at approximately 7.5 Å at all ionic liquid concentrations.

Figures S3e, S3f, and S3g illustrate EG-$[EMIM]^+$, EG-$[BF_4]^-$, and EG-EG RDFs respectively. The height of the first peak is tallest in solvent-anion RDF Figure S3e, followed by the solvent-solvent and solvent-cation RDFs. This suggest stronger solvent-anion and solvent-solvent interactions as compared to solvent-cation interactions. Stronger interaction between the solvent and anion may be attributed to the hydrogen bonding between the F atoms on the anion and H atoms of the hydroxyl groups of EG, as can be visualized from the peaks occurring between 2-3 Å around these atoms in the hydrogen bonding RDFs Figure S3d and Figure S3h. The solvent RDFs do not exhibit significant differences with respect to the peak height, peak position, and the radii of first solvation shell, as a function of ionic liquid concentration. This suggests solvent interactions are not significantly altered due to the presence of ionic liquid.

S2.1.5 Comparison of Nernst-Einstein vs. Einstein Conductivity

Table S6: Ionic Conductivity (S/m) for [EMIM][BF_4] in EG at 298 K

x_{IL}	Avg IC_{NE}	SD IC_{NE}	Avg IC_{EN}	SD IC_{EN}	IC_{EN}/IC_{NE}	Avg IC_{exp}	SD IC_{exp}
1.0	1.33	0.03	1.29	0.11	0.97	1.38	0.027
0.9	1.64	0.04	1.62	0.01	0.98	1.51	0.001
0.8	1.99	0.04	1.47	0.13	0.74	1.57	0.004
0.7	2.32	0.03	1.92	0.10	0.83		
0.6	2.72	0.12	2.16	0.11	0.79	1.89	0.004
0.5	3.08	0.09	2.23	0.22	0.72	2.39	0.005
0.4	3.26	0.09	2.45	0.05	0.75	1.98	0.003
0.3	3.39	0.05	2.48	0.28	0.73	1.64	0.004
0.2	2.93	0.11	2.15	0.08	0.73	1.18	0.002
0.1	1.95	0.10	1.73	0.06	0.88	0.64	0.001

S2.1.6 Ionic Conductivity Estimation using *NPT* and *NVT* Ensembles

Table S7: Comparison of NVT vs. NPT Ensembles for [EMIM][BF_4] in EG at 298 K

	Ionic Conductivity (S/m)		Variation in Cluster Ion Population	
x_{IL}	NVT	NPT	NVT	NPT
1.0	1.33	1.30	1.49	1.50
0.9	1.64	1.64	1.59	1.59
0.8	1.99	1.98	1.69	1.68
0.7	2.32	2.33	1.76	1.77
0.6	2.72	2.69	1.84	1.83
0.5	3.08	2.96	1.90	1.89
0.4	3.26	3.33	**1.93**	**1.93**
0.3	**3.39**	**3.54**	1.86	1.86
0.2	2.93	3.22	1.77	1.73
0.1	1.95	1.85	1.35	1.36

S2.1.7 Cluster Analysis

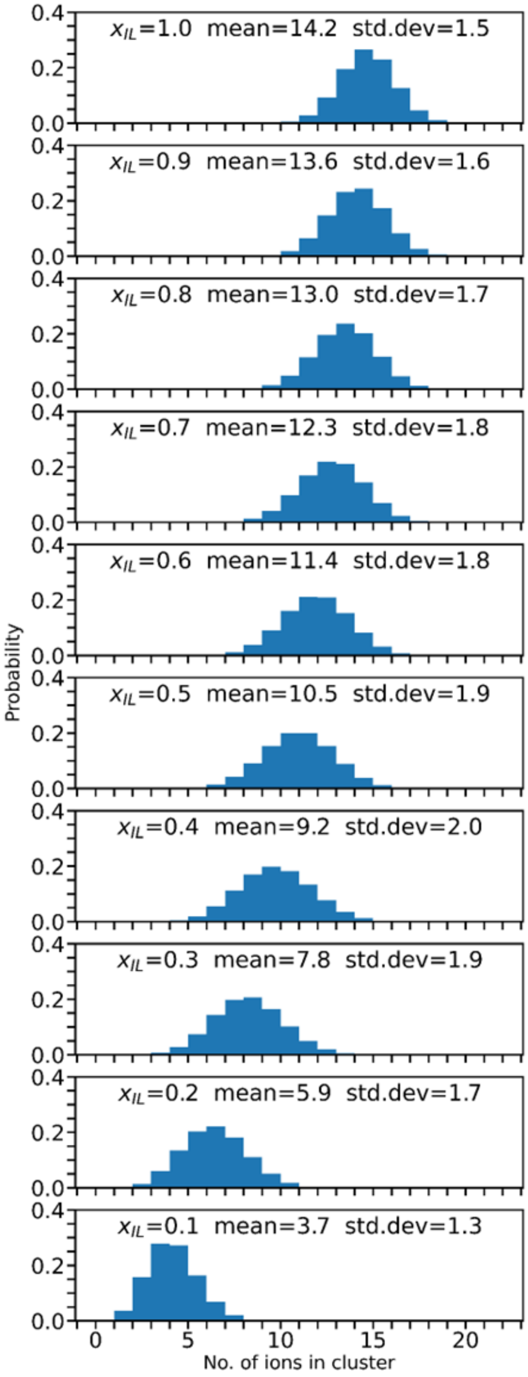

Figure S4: Distribution of ion population in clusters across different ionic liquid concentrations. y-axis shows the average probability of finding clusters with a certain ion population

S2.1.8 Summary of Results

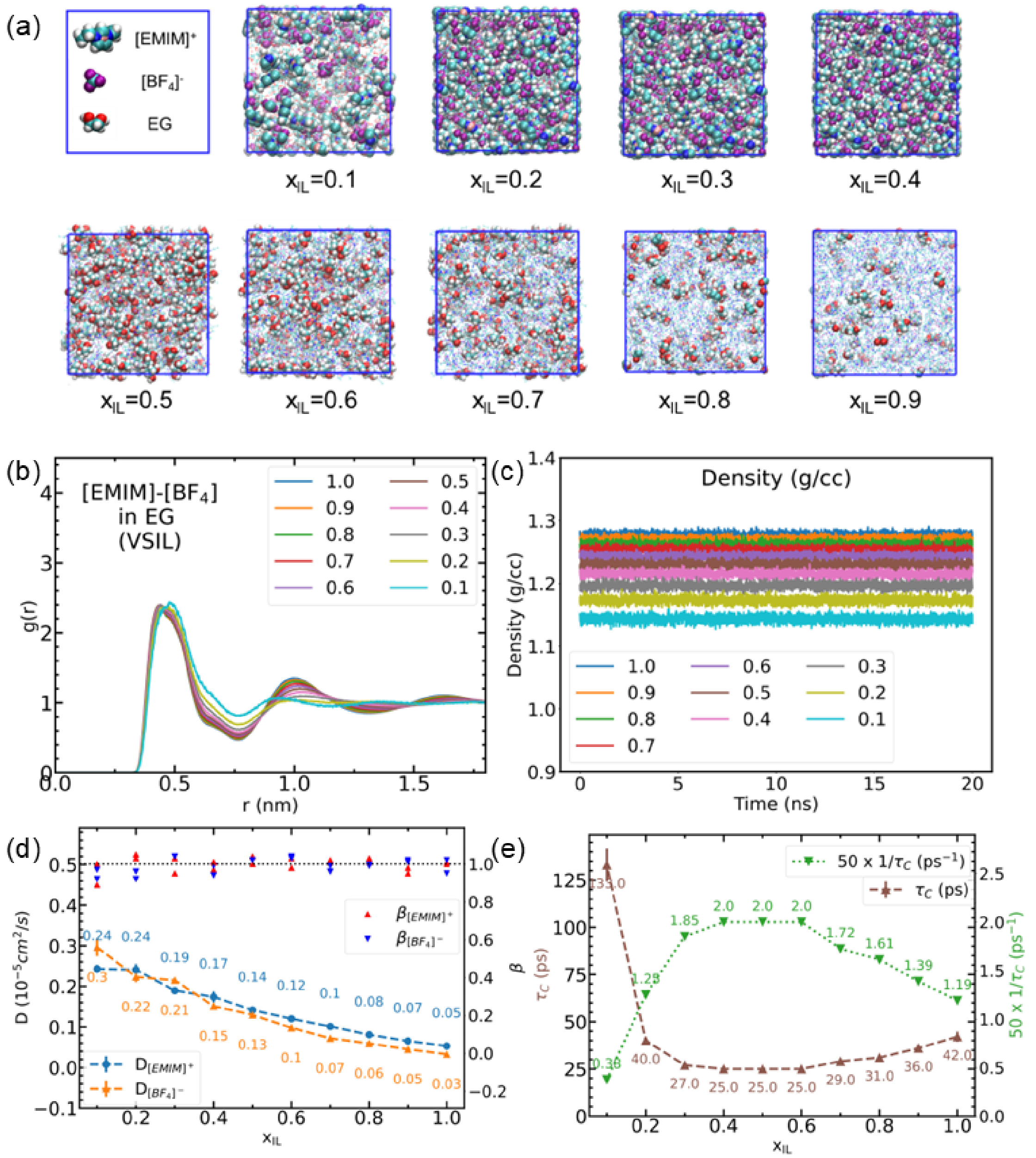

Figure S5: a) Snapshots for [EMIM][BF_4] (VSIL force field) in EG at 298 K. The ions are highlighted in mixtures with $x_{IL} \leq 0.5$ and EG is highlighted at $x_{IL} \geq 0.5$ b) Cation-Anion RDFs c) Bulk density d) Self-diffusion coefficients for ions (primary axis) and corresponding β values (secondary axis) and e) Cage correlation lifetimes (primary axis) and inverse cage correlation lifetimes scaled by 50 for ease of representation (secondary axis) at various compositions with x_{IL} = 0.1 to 1.0

Table S8: Summary of Results for [EMIM][BF_4] in EG at 298 K using VSIL Force Field

x_{IL}	q_+ (e)	q_- (e)	Avg IC_{NE} (S/m)	SD IC_{NE} (S/m)	Avg CIP	SD CIP	Avg τ_C (ps)	SD τ_C (ps)	$x_{IL_{exp}}$	Avg IC_{exp} (S/m)	SD IC_{exp} (S/m)
1	0.8	-0.8	1.33	0.03	13.9	1.48	42	2.7	1	1.38	0.03
0.9	0.8	-0.8	1.64	0.04	13.4	1.57	36	0.3	0.9	1.51	0.00
0.8	0.8	-0.8	1.99	0.04	12.8	1.67	31	0.5	0.8	1.57	0.00
0.7	0.8	-0.8	2.32	0.03	12.1	1.74	29	0.7			0.00
0.6	0.8	-0.8	2.72	0.12	11.3	1.81	25	0.6	0.6	1.89	0.01
0.5	0.8	-0.8	3.08	0.09	10.3	1.87	25	0.3	0.5	2.39	0.00
0.4	0.8	-0.8	3.26	0.09	9.2	1.90	25	0.6	0.4	1.98	0.00
0.3	0.8	-0.8	3.39	0.05	7.7	1.86	27	0.2	0.3	1.64	0.00
0.2	0.8	-0.8	2.93	0.11	5.9	1.73	40	1.2	0.2	1.18	0.00
0.1	0.8	-0.8	1.95	0.10	3.7	1.38	133	8.7	0.1	0.64	0.02

S2.2 0.8*2009IL

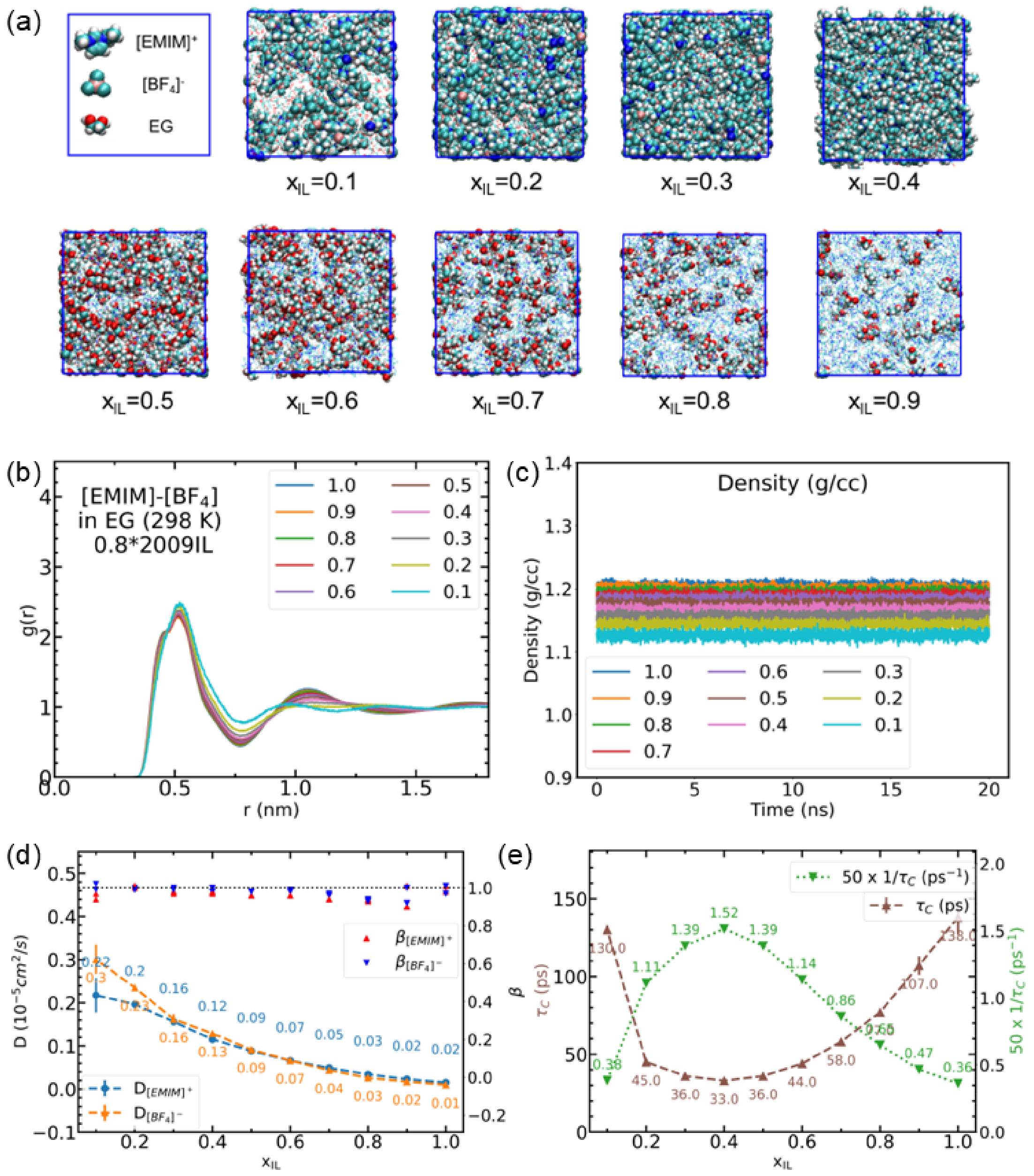

Figure S6: a) Snapshots for [EMIM][BF_4] (0.8*2009IL force field) in EG at 298 K. The ions are highlighted in mixtures with $x_{IL} \leq 0.5$ and EG is highlighted at $x_{IL} \geq 0.5$ b) Cation-Anion RDFs c) Bulk density d) Self-diffusion coefficients for ions (primary axis) and corresponding β values (secondary axis) and e) Cage correlation lifetimes (primary axis) and inverse cage correlation lifetimes scaled by 50 for ease of representation (secondary axis) at various compositions with $x_{IL} = 0.1$ to 1.0

Table S9: Summary of Results for $[EMIM][BF_4]$ in EG at 298 K using 0.8*2009IL Force Field

x_{IL}	q_+ (e)	q_- (e)	Avg IC_{NE} (S/m)	SD IC_{NE} (S/m)	Avg CIP	SD CIP	Avg τ_C (ps)	SD τ_C (ps)	$x_{IL_{exp}}$	Avg IC_{exp} (S/m)	SD IC_{exp} (S/m)
1	0.8	-0.8	0.37	0.01	13.5	1.4	138	11	1	1.38	0.03
0.9	0.8	-0.8	0.57	0.02	13.0	1.5	107	6.0	0.9	1.51	0.00
0.8	0.8	-0.8	0.81	0.03	12.4	1.6	77	0.7	0.8	1.57	0.00
0.7	0.8	-0.8	1.18	0.01	11.8	1.7	58	1.1			
0.6	0.8	-0.8	1.57	0.07	11.0	1.7	44	2.2	0.6	1.89	0.00
0.5	0.8	-0.8	1.95	0.01	10.1	1.8	36	1.8	0.5	2.39	0.01
0.4	0.8	-0.8	2.35	0.03	9.0	1.8	33	0.5	0.4	1.98	0.00
0.3	0.8	-0.8	2.57	0.13	7.6	1.8	36	0.8	0.3	1.64	0.00
0.2	0.8	-0.8	2.65	0.06	5.9	1.7	45	2.3	0.2	1.18	0.00
0.1	0.8	-0.8	1.85	0.17	3.8	1.4	130	2.7	0.1	0.64	0.00

S2.3 DDAP

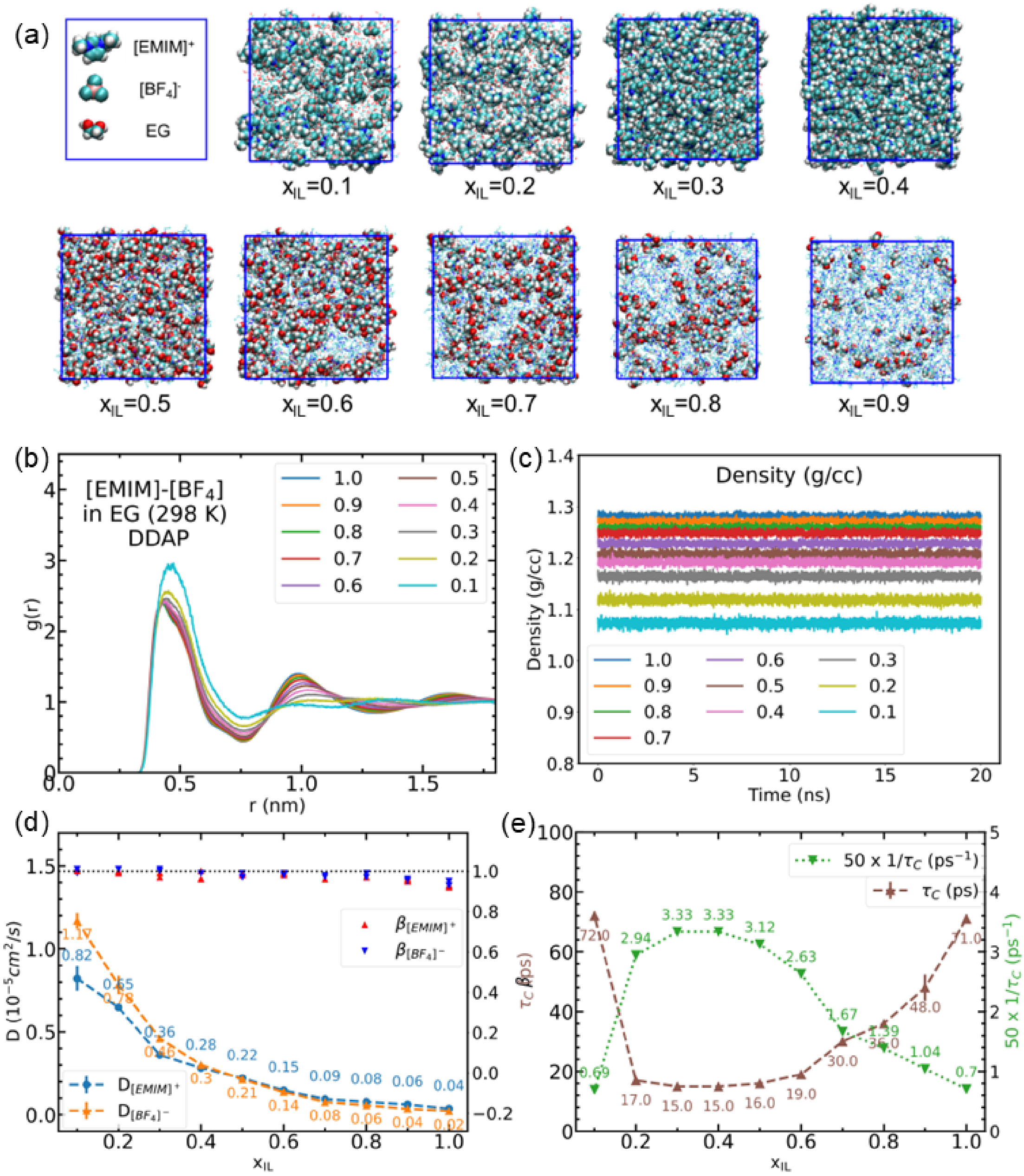

Figure S7: a) Snapshots for [EMIM][BF_4] (DDAP force field) in EG at 298 K. The ions are highlighted in mixtures with $x_{IL} \leq 0.5$ and EG is highlighted at $x_{IL} \geq 0.5$ b) Cation-Anion RDFs c) Bulk density d) Self-diffusion coefficients for ions (primary axis) and corresponding β values (secondary axis) and e) Cage correlation lifetimes (primary axis) and inverse cage correlation lifetimes scaled by 50 for ease of representation (secondary axis) at various compositions with $x_{IL} = 0.1$ to 1.0

Table S10: Summary of Results for [EMIM][BF_4] in EG at 298 K using DDAP derived atomistic charges

x_{IL}	q_+ (e)	q_- (e)	Avg IC_{NE} (S/m)	SD IC_{NE} (S/m)	Avg CIP	SD CIP	Avg τ_C (ps)	SD τ_C (ps)	$x_{IL_{exp}}$	Avg IC_{exp} (S/m)	SD IC_{exp} (S/m)
1	0.80	-0.80	0.93	0.01	13.7	1.5	71	1.4	1	1.38	0.03
0.9	0.80	-0.80	1.45	0.08	13.4	1.6	48	4.4	0.9	1.51	0.00
0.8	0.80	-0.79	1.87	0.07	12.7	1.7	36	0.5	0.8	1.57	0.00
0.7	0.80	-0.79	2.24	0.10	12.0	1.8	30	1.3			
0.6	0.77	-0.76	3.22	0.18	11.1	1.8	19	0.4	0.6	1.89	0.00
0.5	0.77	-0.75	4.38	0.21	10.1	1.9	16	0.2	0.5	2.39	0.01
0.4	0.78	-0.74	5.13	0.27	9.0	1.9	15	0.2	0.4	1.98	0.00
0.3	0.77	-0.73	5.83	0.06	7.5	1.9	15	0.2	0.3	1.64	0.00
0.2	0.73	-0.69	6.80	0.31	5.7	1.7	17	0.5	0.2	1.18	0.00
0.1	0.75	-0.67	5.30	0.12	3.7	1.4	72	1.4	0.1	0.64	0.00

S3 Other Systems

S3.1 [EMIM][TfO] in EG

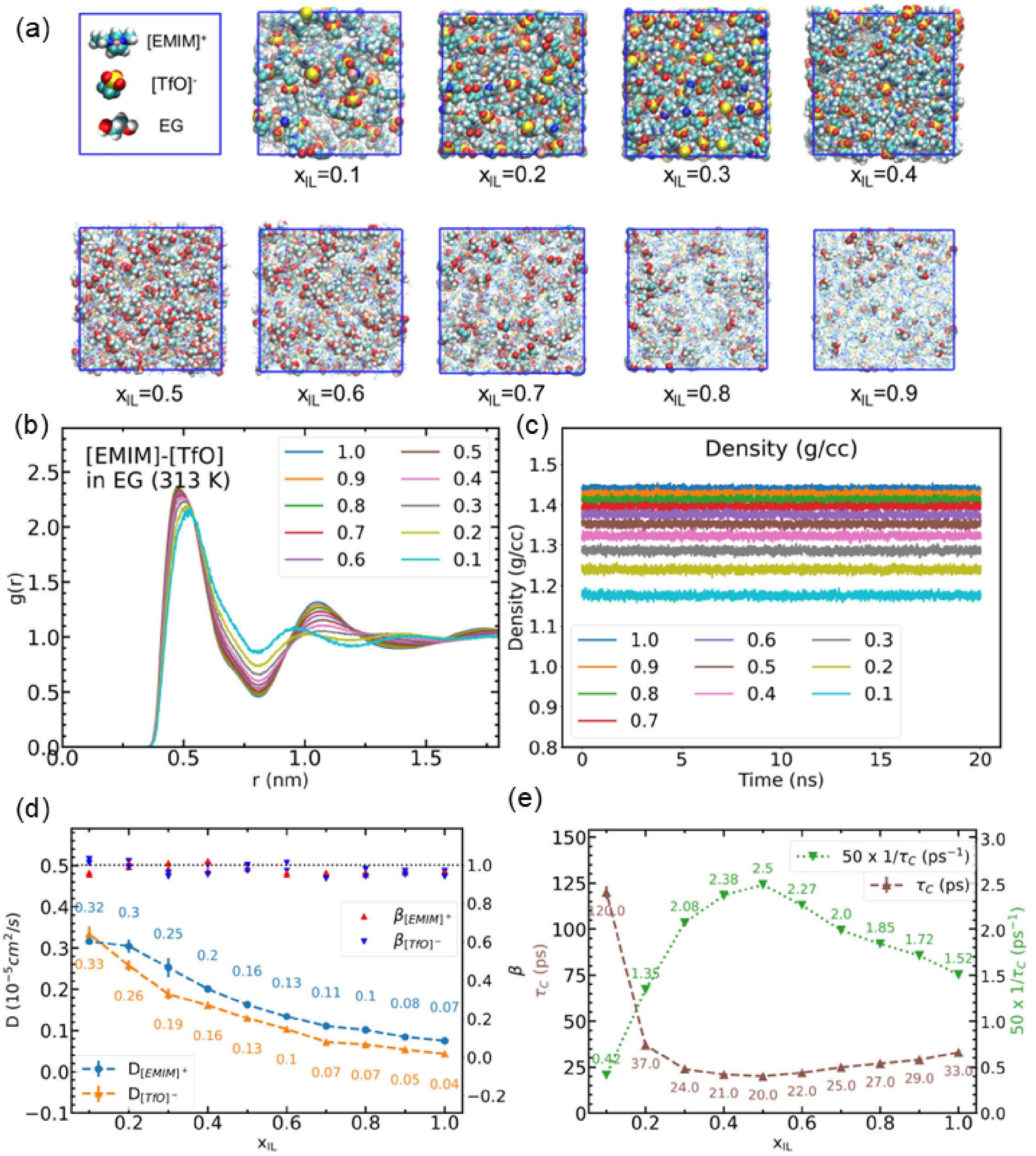

Figure S8: a) Snapshots for [EMIM][TfO] in EG at 313 K. The ions are highlighted in mixtures with $x_{IL} \leq 0.5$ and EG is highlighted at $x_{IL} \geq 0.5$ b) Cation-Anion RDFs c) Bulk density d) Self-diffusion coefficients for ions (primary axis) and corresponding β values (secondary axis) e) Cage correlation lifetimes (primary axis) and inverse cage correlation lifetimes scaled by 50 for ease of representation (secondary axis) at various compositions with $x_{IL} = 0.1$ to 1.0

Table S11: Summary of Results for [EMIM][TfO] in EG at 298 K

x_{IL}	q_+ (e)	q_- (e)	Avg IC_{NE} (S/m)	SD IC_{NE} (S/m)	Avg CIP	SD CIP	Avg τ_C (ps)	SD τ_C (ps)	$x_{IL_{exp}}$	Avg IC_{exp} (S/m)	SD IC_{exp} (S/m)
1	0.8	-0.8	1.49	0.04	14.6	1.43	33	0.2	0.80	1.83	0.07
0.9	0.8	-0.8	1.69	0.04	14.1	1.51	29	0.7	0.59	2.17	0.09
0.8	0.8	-0.8	1.96	0.07	13.5	1.59	27	0.8	0.50	2.28	0.09
0.7	0.8	-0.8	2.06	0.02	13.0	1.64	25	1.7	0.41	2.12	0.09
0.6	0.8	-0.8	2.48	0.05	12.0	1.74	22	0.4	0.30	1.91	0.08
0.5	0.8	-0.8	2.80	0.03	11.1	1.78	20	0.5	0.19	1.51	0.06
0.4	0.8	-0.8	3.09	0.05	9.9	1.79	21	0.4	0.14	1.20	0.05
0.3	0.8	-0.8	3.20	0.25	8.4	1.77	24	0.1	0.09	0.87	0.04
0.2	0.8	-0.8	3.13	0.03	6.5	1.69	37	0.8	0.06	0.56	0.02
0.1	0.8	-0.8	2.13	0.07	4.1	1.43	120	3.5	0.03	0.28	0
0	0.8	-0.8							0.01	0.14	0

S3.2 [EMIM][DCA] in EG

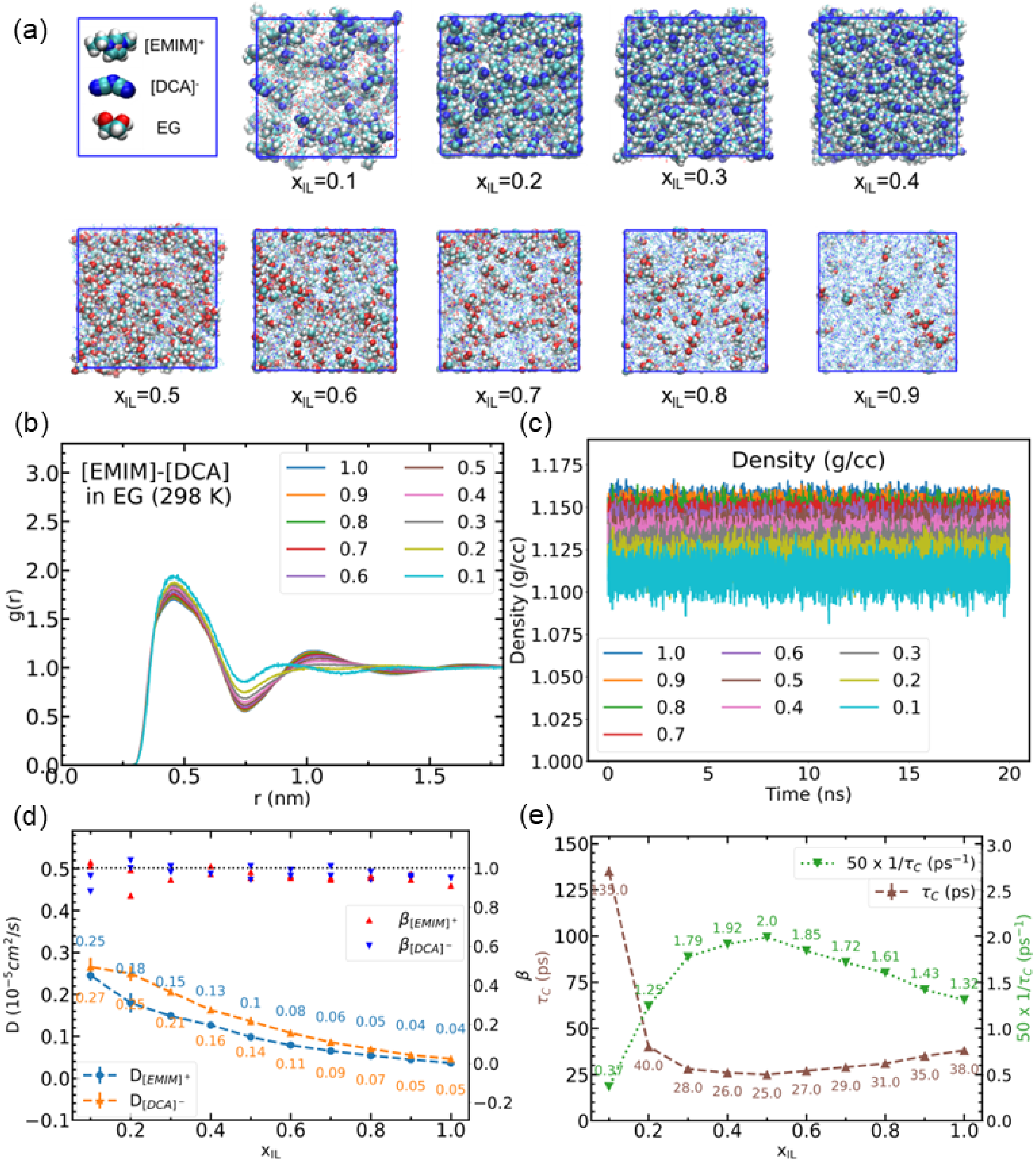

Figure S9: a) Snapshots for [EMIM][DCA] in EG at 298 K. The ions are highlighted in mixtures with $x_{IL} \leq 0.5$ and EG is highlighted at $x_{IL} \geq 0.5$ b) Cation-Anion RDFs c) Bulk density d) Self-diffusion coefficients for ions (primary axis) and corresponding β values (secondary axis) e) Cage correlation lifetimes (primary axis) and inverse cage correlation lifetimes scaled by 50 for ease of representation (secondary axis) at various compositions with x_{IL} = 0.1 to 1.0

Table S12: Summary of Results for [EMIM][DCA] in EG at 298 K

x_{IL}	q_+ (e)	q_- (e)	Avg IC_{NE} (S/m)	SD IC_{NE} (S/m)	Avg CIP	SD CIP	Avg τ_C (ps)	SD τ_C (ps)	$x_{IL_{exp}}$	Avg IC_{exp} (S/m)	SD IC_{exp} (S/m)
1	0.8	-0.8	1.29	0.04	14.1	1.28	38	0.0	1	2.84	0.02
0.9	0.8	-0.8	1.49	0.06	13.6	1.66	35	1.1	0.9	2.98	0.01
0.8	0.8	-0.8	1.76	0.05	13.0	1.73	31	0.9	0.8	3.15	0
0.7	0.8	-0.8	2.03	0.08	12.2	1.81	29	0.3			
0.6	0.8	-0.8	2.33	0.05	11.4	1.85	27	0.7	0.6	3.44	0.01
0.5	0.8	-0.8	2.66	0.18	10.4	1.90	25	0.6	0.5	3.13	0
0.4	0.8	-0.8	2.91	0.14	9.2	1.90	26	1.1	0.4	2.83	0
0.3	0.8	-0.8	2.97	0.03	7.7	1.86	28	0.4	0.3	2.29	0.01
0.2	0.8	-0.8	2.71	0.14	5.9	1.70	40	0.6	0.2	1.63	0.01
0.1	0.8	-0.8	1.85	0.08	3.7	1.38	135	2.5	0.1	0.83	0.03

S3.3 [EMIM][SCN] in EG

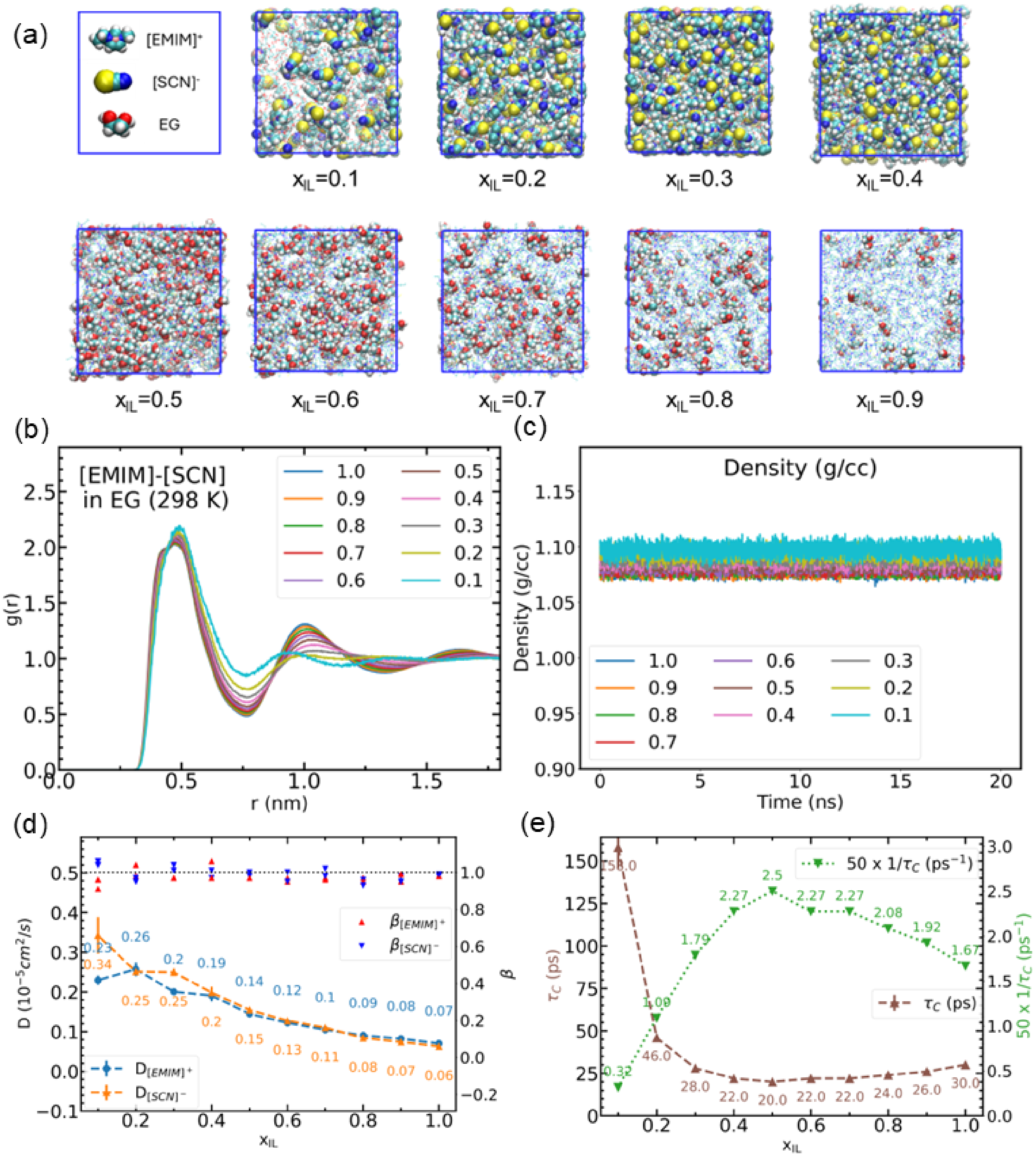

Figure S10: a) Snapshots for [EMIM][SCN] in EG at 298 K. The ions are highlighted in mixtures with $x_{IL} \leq 0.5$ and EG is highlighted at $x_{IL} \geq 0.5$ b) Cation-Anion RDFs c) Bulk density d) Self-diffusion coefficients for ions (primary axis) and corresponding β values (secondary axis) e) Cage correlation lifetimes (primary axis) and inverse cage correlation lifetimes scaled by 50 for ease of representation (secondary axis) at various compositions with $x_{IL} = 0.1$ to 1.0

Table S13: Summary of Results for [EMIM][SCN] in EG at 298 K

x_{IL}	q_+ (e)	q_- (e)	Avg IC_{NE} (S/m)	SD IC_{NE} (S/m)	Avg CIP	SD CIP	Avg τ_C (ps)	SD τ_C (ps)	$x_{IL_{exp}}$	Avg IC_{exp} (S/m)	SD IC_{exp} (S/m)
1	0.8	-0.8	2.04	0.02	14.0	1.48	30	1.0	1	2.22	0.00
0.9	0.8	-0.8	2.30	0.08	13.4	1.57	26	0.5	0.9	2.71	0.00
0.8	0.8	-0.8	2.45	0.06	12.8	1.65	24	0.5	0.8	2.98	0.00
0.7	0.8	-0.8	2.84	0.05	12.1	1.73	22	0.2			
0.6	0.8	-0.8	3.08	0.01	11.3	1.80	22	0.6	0.6	3.37	0.01
0.5	0.8	-0.8	3.37	0.10	10.3	1.85	20	0.1	0.5	2.95	0.01
0.4	0.8	-0.8	3.87	0.10	9.1	1.86	22	0.3	0.4	2.88	0.01
0.3	0.8	-0.8	3.75	0.14	7.7	1.82	28	0.3	0.3	1.97	0.04
0.2	0.8	-0.8	3.20	0.10	5.9	1.71	46	1.2	0.2	1.59	0.00
0.1	0.8	-0.8	2.07	0.15	3.7	1.38	158	12.2	0.1	0.85	0.00

S3.4 [EMIM][BF$_4$] in ACN

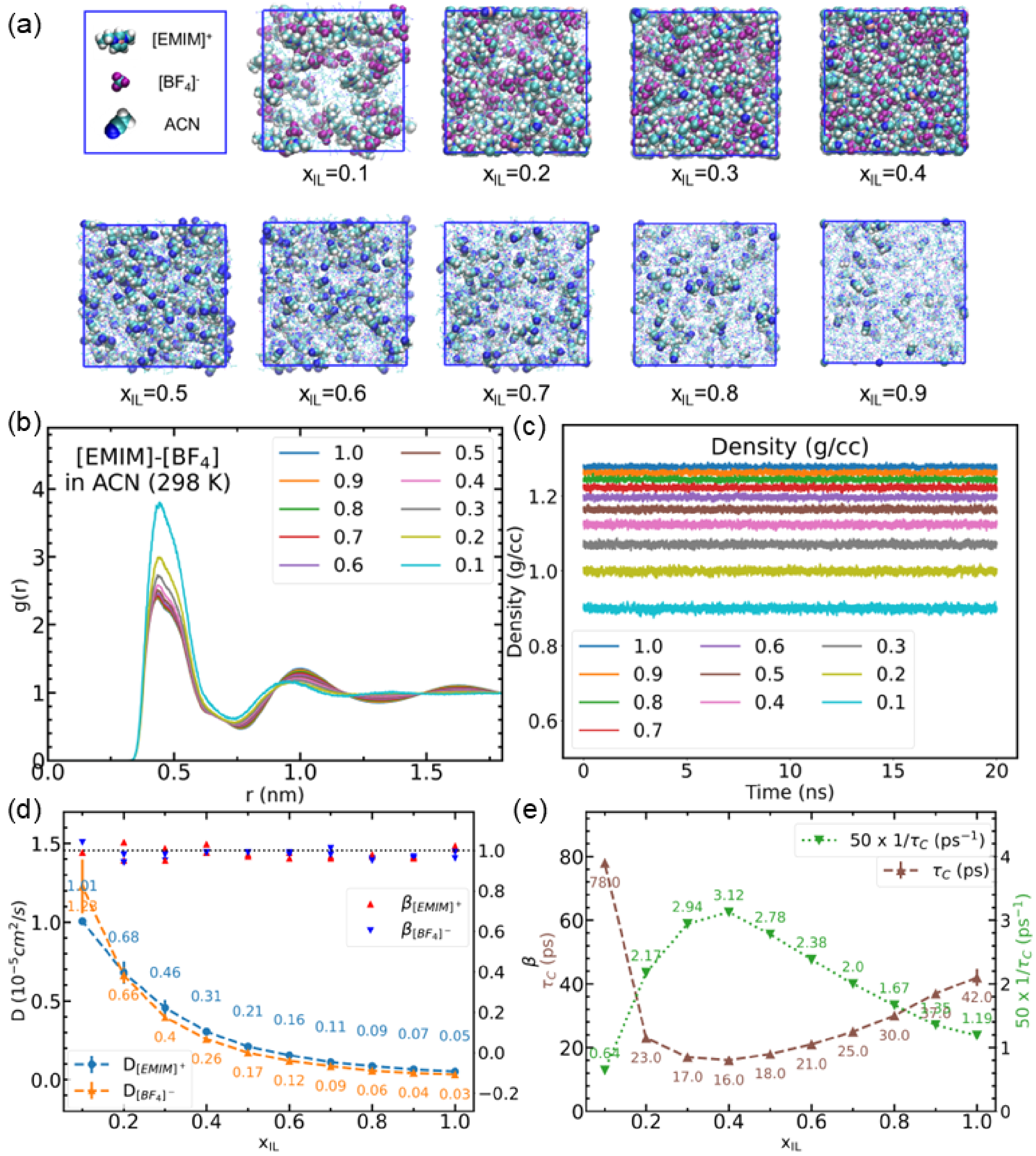

Figure S11: a) Snapshots for [EMIM][BF$_4$] in ACN at 298 K. The ions are highlighted in mixtures with $x_{IL} \leq 0.5$ and EG is highlighted at $x_{IL} \geq 0.5$ b) Cation-Anion RDFs c) Bulk density d) Self-diffusion coefficients for ions (primary axis) and corresponding β values (secondary axis) e) Cage correlation lifetimes (primary axis) and inverse cage correlation lifetimes scaled by 50 for ease of representation (secondary axis) at various compositions with $x_{IL} = 0.1$ to 1.0

Table S14: Summary of Results for [EMIM][BF_4] in ACN at 298 K

x_{IL}	q_+ (e)	q_- (e)	Avg IC_{NE} (S/m)	SD IC_{NE} (S/m)	Avg CIP	SD CIP	Avg τ_C (ps)	SD τ_C (ps)	$x_{IL_{exp}}$	Avg IC_{exp} (S/m)	SD IC_{exp} (S/m)
1	0.8	-0.8	1.33	0.03	13.9	1.5	42	2.7	1.00	1.55	0
0.9	0.8	-0.8	1.63	0.04	13.7	1.6	37	1.0	0.89	1.95	0.02
0.8	0.8	-0.8	2.07	0.03	13.1	1.7	30	0.6	0.76	2.54	0.03
0.7	0.8	-0.8	2.69	0.08	12.4	1.7	25	0.4	0.65	3.20	0.03
0.6	0.8	-0.8	3.51	0.04	11.7	1.8	21	0.1	0.49	4.44	0.04
0.5	0.8	-0.8	4.47	0.15	10.8	1.9	18	0.5	0.38	5.41	0.05
0.4	0.8	-0.8	5.86	0.26	9.7	2.0	16	0.1	0.28	6.41	0.06
0.3	0.8	-0.8	7.45	0.40	8.3	2.0	17	0.2	0.20	6.91	0.07
0.2	0.8	-0.8	8.90	0.70	6.7	2.0	23	0.3	0.14	6.94	0.07
0.1	0.8	-0.8	8.50	0.71	4.6	1.7	78	1.0	0.10	6.50	0.07
									0.05	4.94	0.05

S3.5 [EMIM][BF_4] in EOH

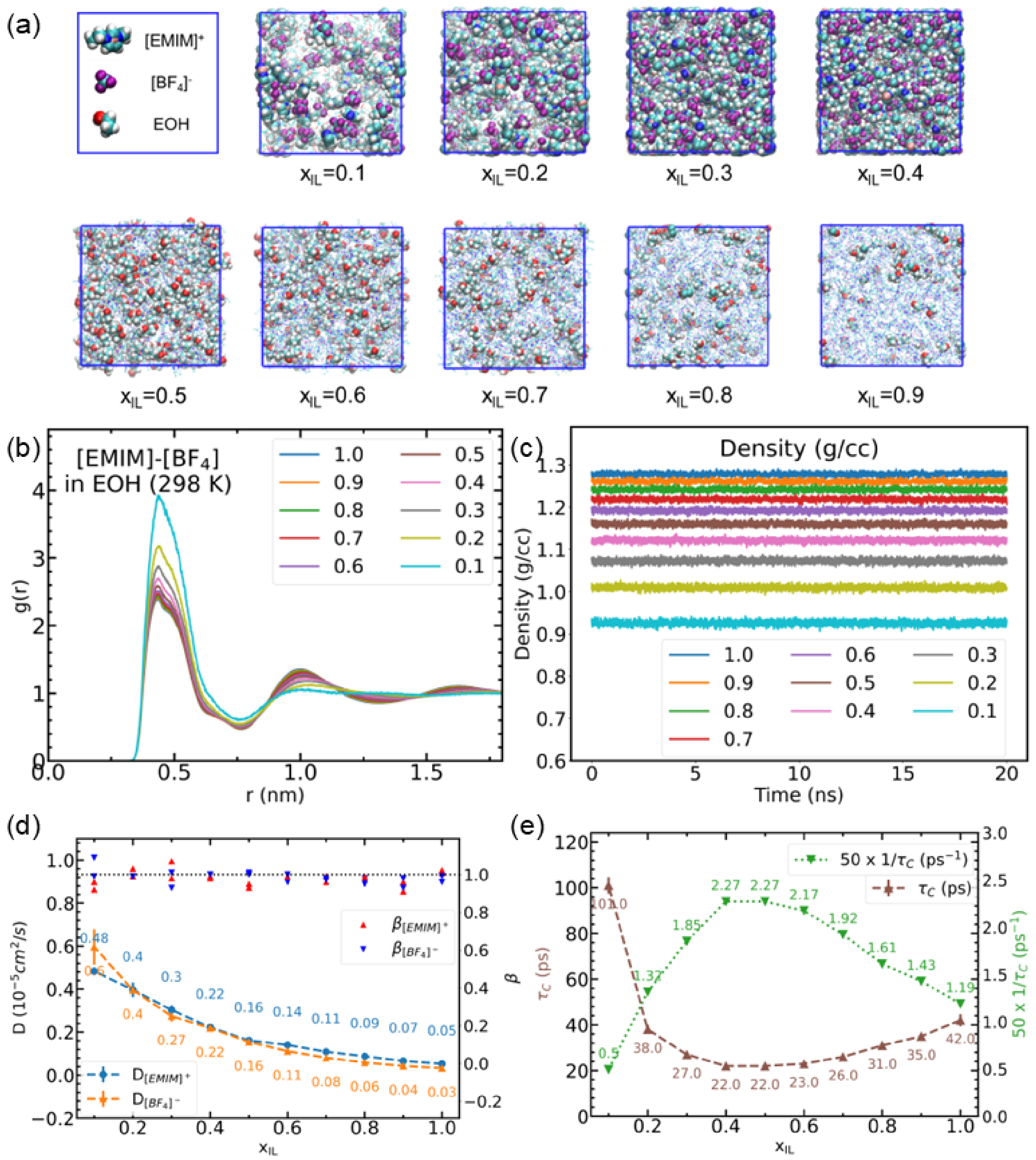

Figure S12: a) Snapshots for [EMIM][BF_4] in EOH at 298 K. The ions are highlighted in mixtures with $x_{IL} \leq 0.5$ and EG is highlighted at $x_{IL} \geq 0.5$ b) Cation-Anion RDFs c) Bulk density d) Self-diffusion coefficients for ions (primary axis) and corresponding β values (secondary axis) e) Cage correlation lifetimes (primary axis) and inverse cage correlation lifetimes scaled by 50 for ease of representation (secondary axis) at various compositions with x_{IL} = 0.1 to 1.0

Table S15: Summary of Results for [EMIM][BF_4] in EOH at 298 K

x_{IL}	q_+ (e)	q_- (e)	Avg IC_{NE} (S/m)	SD IC_{NE} (S/m)	Avg CIP	SD CIP	Avg τ_C (ps)	SD τ_C (ps)	$x_{IL_{exp}}$	Avg IC_{exp} (S/m)	SD IC_{exp} (S/m)
1	0.8	-0.8	1.33	0.03	13.9	1.48	42	2.7	1.00	1.63	
0.9	0.8	-0.8	1.62	0.07	13.6	1.60	35	1.3	0.93	1.91	
0.8	0.8	-0.8	2.08	0.03	13.0	1.72	31	1.3	0.90	2.06	
0.7	0.8	-0.8	2.55	0.08	12.3	1.82	26	1.3	0.84	2.29	
0.6	0.8	-0.8	3.16	0.08	11.5	1.93	23	0.6	0.76	2.65	
0.5	0.8	-0.8	3.61	0.06	10.6	2.01	22	0.5	0.68	2.98	
0.4	0.8	-0.8	4.45	0.12	9.4	2.08	22	0.7	0.61	3.23	
0.3	0.8	-0.8	4.87	0.25	8.1	2.10	27	0.3	0.59	3.28	
0.2	0.8	-0.8	5.02	0.30	6.4	1.99	38	2.0	0.57	3.26	
0.1	0.8	-0.8	3.91	0.36	4.3	1.69	101	3.6	0.55	3.47	

S3.6 [BMIM][BF_4] in EG

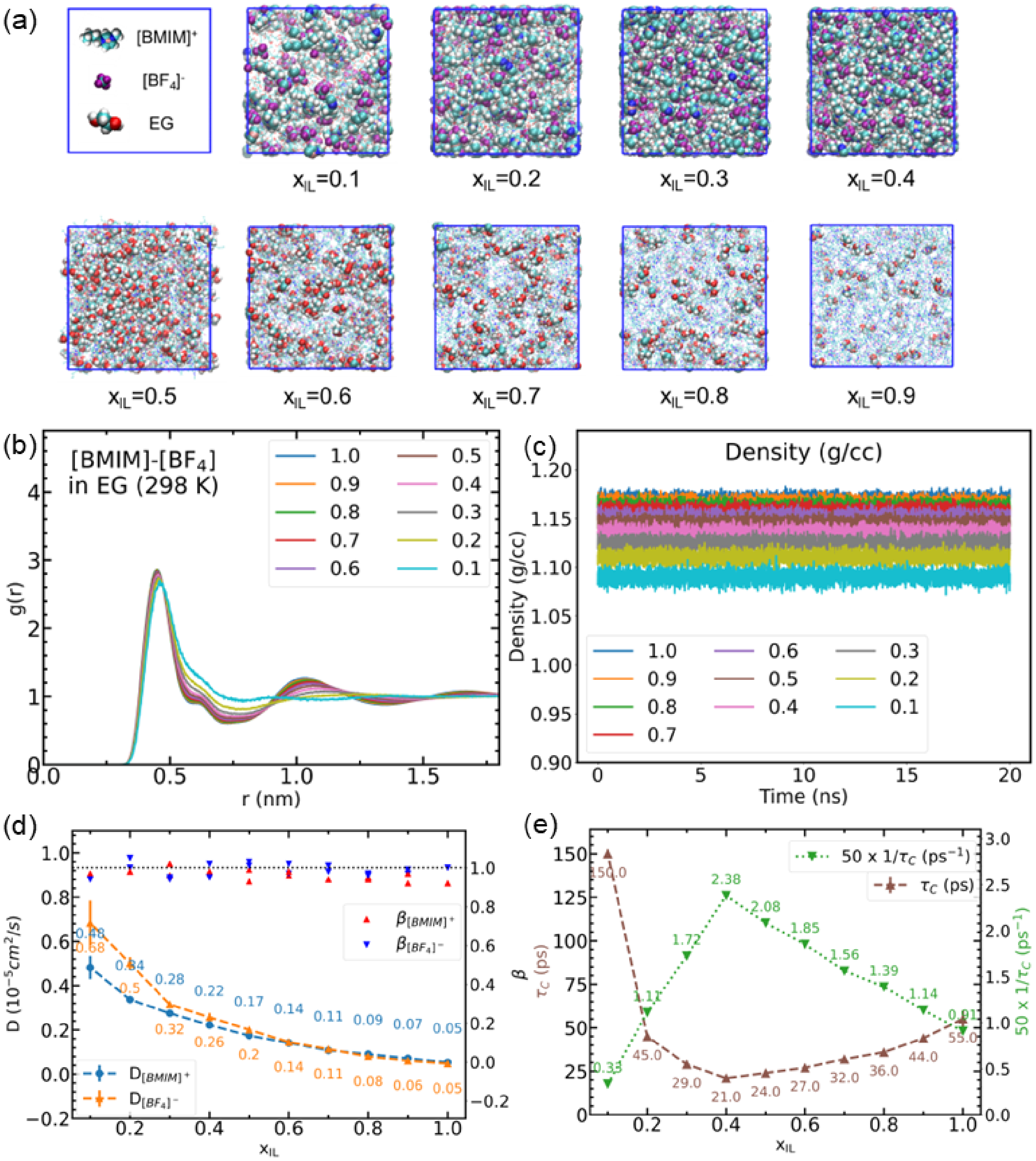

Figure S13: a) Snapshots for [BMIM][BF_4] in EG at 298 K. The ions are highlighted in mixtures with $x_{IL} \leq 0.5$ and EG is highlighted at $x_{IL} \geq 0.5$ b) Cation-Anion RDFs c) Bulk density d) Self-diffusion coefficients for ions (primary axis) and corresponding β values (secondary axis) e) Cage correlation lifetimes (primary axis) and inverse cage correlation lifetimes scaled by 50 for ease of representation (secondary axis) at various compositions with x_{IL} = 0.1 to 1.0

Table S16: Summary of Results for $[BMIM][BF_4]$ in EG at 298 K

x_{IL}	q_+ (e)	q_- (e)	Avg IC_{NE} (S/m)	SD IC_{NE} (S/m)	Avg CIP	SD CIP	Avg τ_C (ps)	SD τ_C (ps)	$x_{IL_{exp}}$	Avg IC_{exp} (S/m)	SD IC_{exp} (S/m)
1	0.8	-0.8	1.12	0.03	11.1	1.60	55	4.9	1.00	0.24	0.00
0.9	0.8	-0.8	1.43	0.05	10.7	1.63	44	0.3			
0.8	0.8	-0.8	1.77	0.05	10.3	1.66	36	1.0			
0.7	0.8	-0.8	2.18	0.04	9.8	1.70	32	1.1	0.65	0.58	0.01
0.6	0.8	-0.8	2.65	0.09	9.2	1.72	27	0.4			
0.5	0.8	-0.8	3.20	0.09	8.5	1.73	24	0.6			
0.4	0.8	-0.8	3.67	0.12	7.6	1.73	21	0.8	0.44	0.77	0.01
0.3	0.8	-0.8	3.85	0.14	6.6	1.69	29	0.2	0.36	0.73	0.01
0.2	0.8	-0.8	4.21	0.20	5.2	1.58	45	0.3	0.18	0.63	0.00
0.1	0.8	-0.8	3.48	0.46	3.4	1.29	150	2.5			

References

(1) Doherty, B.; Zhong, X.; Acevedo, O. Virtual site OPLS force field for imidazolium-based ionic liquids. *The Journal of Physical Chemistry B* **2018**, *122*, 2962–2974.

(2) Doherty, B.; Zhong, X.; Gathiaka, S.; Li, B.; Acevedo, O. Revisiting OPLS force field parameters for ionic liquid simulations. *Journal of chemical theory and computation* **2017**, *13*, 6131–6145.

(3) Sambasivarao, S. V.; Acevedo, O. Development of OPLS-AA force field parameters for 68 unique ionic liquids. *Journal of chemical theory and computation* **2009**, *5*, 1038–1050.

(4) Blöchl, P. Electrostatic decoupling of periodic images of plane-wave-expanded densities and derived atomic point charges. *The Journal of chemical physics* **1995**, *103*, 7422–7428.

(5) Dodda, L. S.; Vilseck, J. Z.; Tirado-Rives, J.; Jorgensen, W. L. 1.14* CM1A-LBCC: localized bond-charge corrected CM1A charges for condensed-phase simulations. *The Journal of Physical Chemistry B* **2017**, *121*, 3864–3870.

(6) Dodda, L. S.; Cabeza de Vaca, I.; Tirado-Rives, J.; Jorgensen, W. L. LigParGen web server: an automatic OPLS-AA parameter generator for organic ligands. *Nucleic acids research* **2017**, *45*, W331–W336.

(7) Jorgensen, W. L.; Tirado-Rives, J. Potential energy functions for atomic-level simulations of water and organic and biomolecular systems. *Proceedings of the National Academy of Sciences* **2005**, *102*, 6665–6670.

(8) Doherty, B.; Acevedo, O. OPLS force field for choline chloride-based deep eutectic solvents. *The Journal of Physical Chemistry B* **2018**, *122*, 9982–9993.

(9) Noda, A.; Hayamizu, K.; Watanabe, M. Pulsed-gradient spin- echo 1H and 19F NMR ionic diffusion coefficient, viscosity, and ionic conductivity of non-chloroaluminate room-temperature ionic liquids. *The Journal of Physical Chemistry B* **2001**, *105*, 4603–4610.

(10) McDaniel, J. G.; Son, C. Y. Ion correlation and collective dynamics in BMIM/BF4-based organic electrolytes: From dilute solutions to the ionic liquid limit. *The Journal of Physical Chemistry B* **2018**, *122*, 7154–7169.
